# Supplementary material for: Monitoring of Water Quality, Antibiotic Residues, and Antibiotic-Resistant Escherichia coli in the Kshipra River in India over a 3-Year Period
Source: Int J Environ Res Public Health. 2020 Oct 22;17(21):7706. doi: 10.3390/ijerph17217706 (PMC7659961; doi:10.3390/ijerph17217706)
Supplement: Supplementary file 1 [file ijerph-17-07706-s001.pdf]

Supplementary Data

Monitoring of water quality, antibiotic residues and antibiotic resistant *Escherichia coli* in the Kshipra river in India over a 3-year period

Nada Hanna [1,*,†], Manju Purohit [1,2,*,†], Vishal Diwan [1,3,4,†], Salesh P. Chandran [5], Emilia Riggi [6], Vivek Parashar [3], Ashok J. Tamhankar [1,7,‡] and Cecilia Stålsby Lundborg [1,‡]

[1] Department of Global Public Health, Health Systems and Policy (HSP): Medicines Focusing Antibiotics, Karolinska Institutet, 171 77 Stockholm, Sweden; vishal.diwan@ki.se (V.D.); ejetee@gmail.com (A.J.T.); Cecilia.Stalsby.Lundborg@ki.se (C.S.L.)
[2] Department of Pathology, R.D. Gardi Medical College, Ujjain 456006, India
[3] Department of Public Health and Environment, R.D. Gardi Medical College, Ujjain 456006, India; vivek.p@rdgmc.edu.in
[4] ICMR—National Institute for Research in Environmental Health, Bhopal 462030, India
[5] HLL Lifecare Ltd., Kharghar Navi, Mumbai 410210, India; saleshp@gmail.com
[6] SSD Epidemiologia screening—CPO, University Hospital 'Cittàdella Salute della Scienza', 10126 Turin, Italy; emilia.riggi@cpo.it
[7] Indian Initiative for Management of Antibiotic Resistance, Department of Environmental Medicine, R.D. Gardi Medical College, Ujjain 456006, India
* Correspondence: nada.hanna@ki.se (N.H.); manju.purohit@ki.se (M.P.)
† Shared first authorship.
‡ Shared last authorship.

Table S1. Description of sampling sites.

**Sampling Point No 1: Kshipra (Upstream Triveni,** 23^0 07′42.516″ N latitude and 75^0 47′ 47.513″ E longitude**):** This is located on the river Kshipra where river Kshipra enters the Ujjain city. This site is situated at upstream of confluence of river Kshipra and river Khan. The maximum depth at this site is about 2 to 3 meter with weed infested banks. Flood plain area of right and left banks is dominated by agricultural activities.

**Sampling Point No 2: Khan (Upstream Triveni,** 23^{0} 07′29.066″ N latitude and 75^{0} 47′ 31.957″ E longitude**):** This is located on the river Khan which brings the domestic as well as industrial wastewater of Indore city, a major town of the region. This site is situated at upstream of confluence of river Kshipra and river Khan. Flood plain area of right and left banks is dominated by agricultural activities.
**Sampling Point No 3: Kshipra (Downstream Triveni,** 23^{0} 07′49.183″ N latitude and 75^{0} 47′ 25.408″ E**):** This is located in close proximity to Dhediya village stop-dam on river Kshipra. This site is situated at downstream of river Kshipra and river Khan confluence. Anthropogenic activities on special mass gatherings are common features at this site. At this sampling site maximum depth of water column is about 4 to 4.5 meter and varies continuously due to stop dam. Flood plain area of right and left banks is dominated by agricultural activities.
**Sampling Point No 4: Ramghat (**23^{0} 11′21.930″ N latitude and 75^{0} 45′ 54.180″ E longitude**):** This is located at the centre of the Ujjain city. This site is the most important religious site and receives maximum number of devotees. Both banks are used for bathing, washing and boating activities all the year round and more often during mass-bathing occasions. The maximum depth at this site is about 2 to 3 meter.
**Sampling Point No 5: Mangalnath (**23^{0} 13′12.923″ N latitude and 75^{0} 47′ 07.045″ E longitude**):** This is located near the ancient Mangalnath temple situated in the right bank of the river. This site is famous for astronomical studies. Anthropogenic activities such as bathing and washing or offering of flower, ashes and coconut by pilgrims is common feature of this site. In the upstream zone of this sampling site two or three small nallas (open drains) join river Kshipra on the left bank which bring the domestic and nearby industry waste of the Ujjain city. The maximum depth (except in case of the height of water at this site is about 5 to 6 meter.
**Sampling Point No 6: Siddhvat (**23^{0} 13′49.765″ N latitude and 75^{0} 46′ 47.162″ E longitude**):** This is located near the ancient Siddhvat gnat situated at the left bank of river. Siddhavat of Ujjain holds a special place for all Hindu pilgrims. Post-funeral rites are performed here. Here Pilgrims offer to the river ashes after funeral and other religious material like flowers, coconut, wheat flour and rice. Khilchipur nalla (an open drain) joins river Kshipra in the upstream of this sampling site on the right bank which brings

the domestic and Industrial waste from eastern part of the Ujjain city and agriculture waste. The maximum depth of water at this site is about 4 to 5 meter.
**Sampling Point No 7: Kaliyadeh (**23^0 14′39.706″ N latitude and 75^0 46′ 55.817″ E longitude)**:** This is the last point before river exits from Ujjain city. Flood plain area of right and left banks is dominated by agricultural activities. The maximum depth at this site is 0.4 to 0.8 meter.

Table S2. The primers and PCR strategy.

PRIMER GENE NAME	SEQUENCE	Cycle plan	PCR Strategy
SUL 1	F5′-TTC GGC ATT CTG AAT CTC AC-3′<br>R5′-ATG ATC TAA CCC TCG GTC TC-3′	94˚C 3 Mints<br>94˚C 30 Secs<br>60˚C<br>40 Secs<br>72˚C<br>50 Secs<br>72˚C<br>7 Mints<br>30 cycles	Single
SUL 2	5′-CGG CAT CGT CAA CAT AAC C-3′<br>5′-GTG TGC GGA TGA AGT CAG-3′		Single
CTXM 1	F5′-TTA GGA ART GTG CCG CTG YA-3′<br>R5′-CGA TAT CGT TGGTGG TRCCAT-3′		CTXM 1,2,9 Multiplex
CTXM 2	F5′-CGT TAA CGG CAC GAT GAC-3′<br>R5′-CGA TAT CGT TGG TGGTRCCAT-3′		
CTXM 9	F5′-TCA AGC CTG CCG ATC TGG T-3′<br>R5′-TGA TTC TCG CCG CTG AAG-3′		
TEM	F5′-CAT TTC CGT GTCGCC CTTATTC-3′<br>R5′-CGT TCATCCATA GTTGCCTGAC-3′		Single
SHV	F5′-AGC CGCTTGAGC AAA TTAAAC-3′<br>R5′-ATC CCGCAGATA AATCAC CAC-3′		Single
qnr A	5′-ATT TCT CAC GCC AGG ATT TG-3′<br>R5′-GATCGGCAA AGG TTA GGT CA-3′		Multiplex
qnr B	F5′-GATCGTGAA AGC CAGAAA GG-3′<br>R5′-ATG AGC AAC GATGCC TGG TA-3′		
qnr S	F5′-GCA AGT TCATTGAACAGG GT-3′<br>R5′-TCT AAA CCGTCGAGTTCGGCG-3′		

NDM	F5′-GGT TTG GCGATCTGG TTTTC-3′<br>R5′-CGG AATGGC TCATCACGA TC-3′		Single
VIM	F5′-GAT GGT GTT TGG TCG CAT A-3′<br>R5′-CGA ATG CGC AGC ACC AG-3′		Single
E. coli	F5′-CACATTCTC ATC GCGGTCGACC-3′<br>R5′-CGTGGATCAGAGAAACTT TCG CAT TGT AGG-3′	94°C 3 Mints<br>94°C 30 Secs<br>57°C<br>40 Secs<br>72°C<br>50 Secs<br>72°C<br>7 Mints<br>35 cycles	Single
ChuA	5′-GACGAACCAACGGTCAGGAT-3′<br>5′-TGCCGCCAGTACCAAAGACA-3′	94°C<br>5 Mints, 94°C<br>30 Secs, 55°C<br>30 Secs, 72°C<br>30 Secs, 72°C<br>7 mints 30 cycles	multiplex
YjaA	5′-TGAAGTGTCAGGAGACGCTG-3 5′-ATGGAGAATGCGTTCCTCAAC-3		
TspE4C2	5′-GAGTAATGTCGGGGCATTCA-3′ 5′-CGCGCCAACAAAGTATTACG-3′		

Table S3. Number of samples with antibiotic residues detected in various seasons in water and sediments of Kshipra river in India over a 3-year period.

**WATER**												
**Antibiotic**	**First year**				**Second year**				**Third year**			
	**Summer (N=84)**	**Rain (N=84)**	**Autumn (N=78)**	**Winter (N=84)**	**Summer (N=84)**	**Rain (N=84)**	**Autumn (N=85)**	**Winter (N=84)**	**Summer (N=84)**	**Rain (N=84)**	**Autumn (N=84)**	**Winter (N=84)**
	**n**	**n**	**n**	**n**	**n**	**n**	**n**	**n**	**n**	**n**	**n**	**n**
Ceftriaxone	0	0	0	0	0	0	0	0	0	NA	NA	NA
Ciprofloxacin	0	0	0	0	0	0	0	0	0	0	0	NA
Norfloxacin	0	0	66	0	0	0	0	0	0	NA	NA	NA
Ofloxacin	0	0	57	0	0	0	0	0	12	NA	NA	NA
Metronidazole	0	0	0	0	0		0	0	0	NA	NA	NA
Sulfamethoxazole	84	24	78	84	84	78	85	24	0	0	0	NA
**Total Residual Antibiotics as β-lactam**	**n**	**n**	**n**	**n**	**n**	**n**	**n**	**n**	**n**	**n**	**n**	**n**
Present (>5ppb)	42	0	0	0	0	0	0	0	0	0	0	0

SEDIMENTS												
**Antibiotic**	**First year**				**Second year**				**Third year**			
	**Summer (N=0)**	**Rain (N=34)**	**Autumn (N=29)**	**Winter (N=41)**	**Summer (N=43)**	**Rain (N=42)**	**Autumn (N=43)**	**Winter (N=42)**	**Summer (N=42)**	**Rain (N=42)**	**Autumn (N=42)**	**Winter (N=42)**
	**n**	**n**	**n**	**n**	**n**	**n**	**n**	**n**	**n**	**n**	**n**	**n**
Ceftriaxone	Not collected	0	0	0	0	0	0	0	0	NA	NA	NA
Ciprofloxacin	Not collected	0	0	0	0	0	0	0	0	0	0	NA
Norfloxacin	Not collected	0	0	0	0	0	0	0	0	NA	NA	NA
Ofloxacin	Not collected	0	0	5	0	0	0	0	0	NA	NA	NA
Metronidazole	Not collected	0	0	0	0	0	0	0	0	NA	NA	NA
Sulfamethoxazole	Not collected	0	0	6	0	0	0	0	0	0	0	NA
**Total Residual Antibiotics as β-lactam**	**n**	**n**	**n**	**n**	**n**	**n**	**n**	**n**	**n**	**n**	**n**	**n**
Present (>5ppb)	Not collected	19	0	0	0	0	0	0	0	0	0	0

Abbreviations: N: total number of samples collected, n: number of samples with antibiotic residues detected, NA: missing.

Table S4. Concentrations of antibiotic residues measured in waters of the Kshipra river in India in various seasons and at various sites over a 3-year period.

SITE 1												
**Antibiotics**	**First year**				**Second year**				**Third year**			
	**Summer**	**Rain**	**Autumn**	**Winter**	**Summer**	**Rain**	**Autumn**	**Winter**	**Summer**	**Rain**	**Autumn**	**Winter**
	**Mean (µg/L)**	**Mean (µg/L)**	**Mean (µg/L)**	**Mean (µg/L)**	**Mean (µg/L)**	**Mean (µg/L)**	**Mean (µg/L)**	**Mean (µg/L)**	**Mean (µg/L)**	**Mean (µg/L)**	**Mean (µg/L)**	**Mean (µg/L)**
Ceftriaxone	BDL	BDL	BDL	BDL	BDL	BDL	BDL	BDL	BDL	NA	NA	NA
Ciprofloxacin	BDL	BDL	BDL	BDL	BDL	BDL	BDL	BDL	BDL	BDL	BDL	NA
Norfloxacin	BDL	BDL	0.81	BDL	BDL	BDL	BDL	BDL	BDL	NA	NA	NA
Ofloxacin	BDL	BDL	0.64	BDL	BDL	BDL	BDL	BDL	BDL	NA	NA	NA
Metronidazole	BDL	BDL	BDL	BDL	BDL	0.23	BDL	BDL	BDL	NA	NA	NA
Sulfamethoxazole	0.31	BDL	1.05	2.46	0.42	0.2	2.6	0.56	BDL	BDL	BDL	NA
**Total Residual Antibiotics as β-lactam**	**n (%)**	**n (%)**	**n (%)**	**n (%)**	**n (%)**	**n (%)**	**n (%)**	**n (%)**	**n (%)**	**n (%)**	**n (%)**	**n (%)**
Present (>5ppb)	6 (50)	0 (0)	0 (0)	0 (0)	0 (0)	0 (0)	0 (0)	0 (0)	0 (0)	0 (0)	0 (0)	0 (0)
**SITE 2**												
**Antibiotics**	**First year**				**Second year**				**Third year**			
	**Summer**	**Rain**	**Autumn**	**Winter**	**Summer**	**Rain**	**Autumn**	**Winter**	**Summer**	**Rain**	**Autumn**	**Winter**
	**Mean (µg/L)**	**Mean (µg/L)**	**Mean (µg/L)**	**Mean (µg/L)**	**Mean (µg/L)**	**Mean (µg/L)**	**Mean (µg/L)**	**Mean (µg/L)**	**Mean (µg/L)**	**Mean (µg/L)**	**Mean (µg/L)**	**Mean (µg/L)**
Ceftriaxone	BDL	BDL	BDL	BDL	BDL	BDL	BDL	BDL	BDL	NA	NA	NA
Ciprofloxacin	BDL	BDL	BDL	BDL	BDL	BDL	BDL	BDL	BDL	BDL	BDL	NA
Norfloxacin	BDL	BDL	0.98	BDL	BDL	BDL	BDL	BDL	BDL	NA	NA	NA
Ofloxacin	BDL	BDL	1.23	BDL	BDL	BDL	BDL	BDL	BDL	NA	NA	NA
Metronidazole	BDL	BDL	BDL	BDL	BDL	0.10	BDL	BDL	BDL	NA	NA	NA
Sulfamethoxazole	2.1	0.13	4.66	2.64	0.99	0.3	2.3	BDL	BDL	BDL	BDL	NA
**Total Residual Antibiotics as β-lactam**	**n (%)**	**n (%)**	**n (%)**	**n (%)**	**n (%)**	**n (%)**	**n (%)**	**n (%)**	**n (%)**	**n (%)**	**n (%)**	**n (%)**
Present (>5ppb)	6 (50)	0 (0)	0 (0)	0 (0)	0 (0)	0 (0)	0 (0)	0 (0)	0 (0)	0 (0)	0(0)	0 (0)

SITE 3												
**Antibiotics**	**First year**				**Second year**				**Third year**			
	**Summer**	**Rain**	**Autumn**	**Winter**	**Summer**	**Rain**	**Autumn**	**Winter**	**Summer**	**Rain**	**Autumn**	**Winter**
	**Mean (µg/L)**	**Mean (µg/L)**	**Mean (µg/L)**	**Mean (µg/L)**	**Mean (µg/L)**	**Mean (µg/L)**	**Mean (µg/L)**	**Mean (µg/L)**	**Mean (µg/L)**	**Mean (µg/L)**	**Mean (µg/L)**	**Mean (µg/L)**
Ceftriaxone	BDL	BDL	BDL	BDL	BDL	BDL	BDL	BDL	BDL	NA	NA	NA
Ciprofloxacin	BDL	BDL	BDL	BDL	BDL	BDL	BDL	BDL	BDL	BDL	BDL	NA
Norfloxacin	BDL	BDL	0.83	BDL	BDL	BDL	BDL	BDL	BDL	NA	NA	NA
Ofloxacin	BDL	BDL	0.64	BDL	BDL	BDL	BDL	BDL	BDL	NA	NA	NA
Metronidazole	BDL	BDL	BDL	BDL	BDL	0.11	BDL	BDL	BDL	NA	NA	NA
Sulfamethoxazole	1.36	0.15	1.32	3.11	0.76	BDL	1.6	BDL	BDL	BDL	BDL	NA
**Total Residual Antibiotics as β-lactam**	**n (%)**	**n (%)**	**n (%)**	**n (%)**	**n (%)**	**n (%)**	**n (%)**	**n (%)**	**n (%)**	**n (%)**	**n (%)**	**n (%)**
Present (>5ppb)	12 (100)	0 (0)	0 (0)	0 (0)	0 (0)	0 (0)	0 (0)	0 (0)	0 (0)	0 (0)	0 (0)	0 (0)
**SITE 4**												
**Antibiotics**	**First year**				**Second year**				**Third year**			
	**Summer**	**Rain**	**Autumn**	**Winter**	**Summer**	**Rain**	**Autumn**	**Winter**	**Summer**	**Rain**	**Autumn**	**Winter**
	**Mean (µg/L)**	**Mean (µg/L)**	**Mean (µg/L)**	**Mean (µg/L)**	**Mean (µg/L)**	**Mean (µg/L)**	**Mean (µg/L)**	**Mean (µg/L)**	**Mean (µg/L)**	**Mean (µg/L)**	**Mean (µg/L)**	**Mean (µg/L)**
Ceftriaxone	BDL	BDL	BDL	BDL	BDL	BDL	BDL	BDL	BDL	NA	NA	NA
Ciprofloxacin	BDL	BDL	BDL	BDL	BDL	BDL	BDL	BDL	BDL	BDL	BDL	NA
Norfloxacin	BDL	BDL	0.67	BDL	BDL	BDL	BDL	BDL	BDL	NA	NA	NA
Ofloxacin	BDL	BDL	1.03	BDL	BDL	BDL	BDL	BDL	BDL	NA	NA	NA
Metronidazole	BDL	BDL	BDL	BDL	BDL	0.06	BDL	BDL	BDL	NA	NA	NA
Sulfamethoxazole	1.36	BDL	2.31	0.5	0.87	0.23	1.00	BDL	BDL	BDL	BDL	NA
**Total Residual Antibiotics as β-lactam**	**n (%)**	**n (%)**	**n (%)**	**n (%)**	**n (%)**	**n (%)**	**n (%)**	**n (%)**	**n (%)**	**n (%)**	**n (%)**	**n (%)**
Present (>5ppb)	6 (50)	0 (0)	0 (0)	0 (0)	0 (0)	0 (0)	0 (0)	0 (0)	0 (0)	0 (0)	0 (0)	0 (0)
**SITE 5**												
	**First year**				**Second Year**				**Third year**			
	**Summer**	**Rain**	**Autumn**	**Winter**	**Summer**	**Rain**	**Autumn**	**Winter**	**Summer**	**Rain**	**Autumn**	**Winter**

Antibiotics	Mean (µg/L)	Mean (µg/L)	Mean (µg/L)	Mean (µg/L)	Mean (µg/L)	Mean (µg/L)	Mean (µg/L)	Mean (µg/L)	Mean (µg/L)	Mean (µg/L)	Mean (µg/L)	Mean (µg/L)
Ceftriaxone	BDL	BDL	BDL	BDL	BDL	BDL	BDL	BDL	BDL	NA	NA	NA
Ciprofloxacin	BDL	BDL	BDL	BDL	BDL	BDL	BDL	BDL	BDL	BDL	BDL	NA
Norfloxacin	BDL	BDL	0.64	BDL	BDL	BDL	BDL	BDL	BDL	NA	NA	NA
Ofloxacin	BDL	BDL	1.46	BDL	BDL	BDL	BDL	BDL	BDL	NA	NA	NA
Metronidazole	BDL	BDL	BDL	BDL	BDL	0.23	BDL	BDL	BDL	NA	NA	NA
Sulfamethoxazole	2.06	BDL	2.93	3.47	0.84	0.31	1.1	0.54	BDL	BDL	BDL	NA
**Total Residual Antibiotics as β-lactam**	**n (%)**	**n (%)**	**n (%)**	**n (%)**	**n (%)**	**n (%)**	**n (%)**	**n (%)**	**n (%)**	**n (%)**	**n (%)**	**n (%)**
Present (>5ppb)	6 (50)	0 (0)	0 (0)	0 (0)	0 (0)	0 (0)	0 (0)	0 (0)	0 (0)	0 (0)	0 (0)	0 (0)
**SITE 6**												
**Antibiotics**	**First year**				**Second year**				**Third year**			
	**Summer**	**Rain**	**Autumn**	**Winter**	**Summer**	**Rain**	**Autumn**	**Winter**	**Summer**	**Rain**	**Autumn**	**Winter**
	**Mean (µg/L)**	**Mean (µg/L)**	**Mean (µg/L)**	**Mean (µg/L)**	**Mean (µg/L)**	**Mean (µg/L)**	**Mean (µg/L)**	**Mean (µg/L)**	**Mean (µg/L)**	**Mean (µg/L)**	**Mean (µg/L)**	**Mean (µg/L)**
Ceftriaxone	BDL	BDL	BDL	BDL	BDL	BDL	BDL	BDL	BDL	NA	NA	NA
Ciprofloxacin	BDL	BDL	BDL	BDL	BDL	BDL	BDL	BDL	BDL	BDL	BDL	NA
Norfloxacin	BDL	BDL	0.67	BDL	BDL	BDL	BDL	BDL	BDL	NA	NA	NA
Ofloxacin	BDL	BDL	1.05	BDL	BDL	BDL	BDL	BDL	2.61	NA	NA	NA
Metronidazole	BDL	BDL	BDL	BDL	BDL	0.27	BDL	BDL	BDL	NA	NA	NA
Sulfamethoxazole	1.66	BDL	2.94	2.43	0.71	0.27	1.00	BDL	BDL	BDL	BDL	NA
**Total Residual Antibiotics as β-lactam**	**n (%)**	**n (%)**	**n (%)**	**n (%)**	**n (%)**	**n (%)**	**n (%)**	**n (%)**	**n (%)**	**n (%)**	**n (%)**	**n (%)**
Present (>5ppb)	0(0)	0 (0)	0 (0)	0(0)	0(0)	0 (0)	0 (0)	0 (0)	0 (0)	0 (0)	0 (0)	0 (0)
**SITE 7**												
**Antibiotics**	**First year**				**Second year**				**Third year**			
	**Summer**	**Rain**	**Autumn**	**Winter**	**Summer**	**Rain**	**Autumn**	**Winter**	**Summer**	**Rain**	**Autumn**	**Winter**
	**Mean (µg/L)**	**Mean (µg/L)**	**Mean (µg/L)**	**Mean (µg/L)**	**Mean (µg/L)**	**Mean (µg/L)**	**Mean (µg/L)**	**Mean (µg/L)**	**Mean (µg/L)**	**Mean (µg/L)**	**Mean (µg/L)**	**Mean (µg/L)**
Ceftriaxone	BDL	BDL	BDL	BDL	BDL	BDL	BDL	BDL	BDL	NA	NA	NA
Ciprofloxacin	BDL	BDL	BDL	BDL	BDL	BDL	BDL	BDL	BDL	BDL	BDL	NA

Norfloxacin	BDL	BDL	0.67	BDL	BDL	BDL	BDL	BDL	BDL	NA	NA	NA
Ofloxacin	BDL	BDL	0.85	BDL	BDL	BDL	BDL	BDL	BDL	NA	NA	NA
Metronidazole	BDL	BDL	BDL	BDL	BDL	0.23	BDL	BDL	BDL	NA	NA	NA
Sulfamethoxazole	0.92	BDL	4.02	0.64	0.73	0.38	1.1	BDL	BDL	BDL	BDL	NA
**Total Residual Antibiotics as β-lactam**	**n (%)**	**n (%)**	**n (%)**	**n (%)**	**n (%)**	**n (%)**	**n (%)**	**n (%)**	**n (%)**	**n (%)**	**n (%)**	**n (%)**
Present (>5ppb)	6 (50)	0 (0)	0 (0)	0 (0)	0 (0)	0 (0)	0 (0)	0 (0)	0 (0)	0 (0)	0 (0)	0 (0)

**Antibiotics**	**P-value***
Ceftriaxone	-
Ciprofloxacin	-
Norfloxacin	-
Ofloxacin	<0.0001
Metronidazole	-
Sulfamethoxazole	<0.0001
**Total Residual Antibiotics as β-lactam** Present (>5ppb)	<0.0001

Abbreviations: BDL: below detection limit, NA: missing.

Note: *p-value are extracted from Analysis of Variance (ANOVA).

Table S5. Seasonal pairwise comparison of antibiotic residues in waters of the Kshipra river in India over a 3-year period.

**River water samples**		
**Antibiotics**		
**Comparison**	Ofloxacin	Sulfamethoxazole
Autumn -Rain	<0.0001	<0.0001
Autumn -Summer	<0.0001	<0.0001
Autumn -Winter	<0.0001	<0.0001
Rain -Summer	1.00	<0.0001
Rain -Winter	1.00	<0.0001
Summer -Winter	1.00	<0.0001
Autumn -Rain	1.00	<0.0001
Autumn -Summer	1.00	<0.0001
Autumn -Winter	1.00	<0.0001
Rain -Summer	1.00	<0.0001
Rain -Winter	1.00	0.99
Summer -Winter	1.00	<0.0001
Autumn -Rain	-	1.00
Autumn -Summer	-	1.00
Autumn -Winter	-	-
Rain -Summer	-	1.00
Rain -Winter	-	-
Summer -Winter	-	-

Note: p-value adjusted with Tukey method.

Table S6. Antibiotic resistance patterns in *E. coli* (393 total number of isolates, 97 in first year, 151 second year, 145 third year) from sediment samples of the Kshipra river in India in various seasons and at various sites over a 3-year period.

Antibiotic	First year																				
	Rain							Autumn							Winter						
	Site 1 (N=0)	Site 2 (N=3)	Site 3 (N=5)	Site 4 (N=6)	Site 5 (N=5)	Site 6 (N=6)	Site 7 (N=6)	Site 1 (N=6)	Site 2 (N=3)	Site 3 (N=7)	Site 4 (N=0)	Site 5 (N=5)	Site 6 (N=3)	Site 7 (N=3)	Site 1 (N=5)	Site 2 (N=5)	Site 3 (N=6)	Site 4 (N=6)	Site 5 (N=5)	Site 6 (N=6)	Site 7 (N=6)
	n(%)	n(%)	n(%)	n(%)	n(%)	n(%)	n(%)	n(%)	n(%)	n(%)	n(%)	n(%)	n(%)	n(%)	n(%)	n(%)	n(%)	n(%)	n(%)	n(%)	n(%)
Ampicillin	0(0)	3(100)	0(0)	1(17)	0(0)	1(17)	4(67)	3(50)	0(0)	2(29)	0(0)	1(20)	1(33)	1(33)	0(0)	2(40)	2(33)	0(0)	2(40)	3(50)	1(17)
Cefotaxime	0(0)	3(100)	0(0)	1(17)	1(20)	1(17)	3(50)	2(33)	0(0)	2(29)	0(0)	0(0)	1(33)	1(33)	0(0)	2(40)	1(17)	0(0)	1(20)	3(50)	0(0)
Ceftazidime	0(0)	3(100)	0(0)	1(17)	1(20)	1(17)	2(33)	2(33)	0(0)	2(29)	0(0)	0(0)	0(0)	1(33)	0(0)	0(0)	1(17)	0(0)	1(20)	1(17)	1(17)
Cefepime	0(0)	0(0)	0(0)	0(0)	0(0)	0(0)	1(17)	0(0)	0(0)	0(0)	0(0)	0(0)	0(0)	1(33)	0(0)	0(0)	1(17)	0(0)	1(20)	1(17)	0(0)
Amikacin	0(0)	0(0)	0(0)	0(0)	0(0)	0(0)	0(0)	0(0)	0(0)	0(0)	0(0)	1(20)	0(0)	0(0)	0(0)	0(0)	0(0)	0(0)	0(0)	0(0)	1(17)
Gentamicin	0(0)	0(0)	0(0)	0(0)	0(0)	0(0)	0(0)	0(0)	0(0)	0(0)	0(0)	0(0)	0(0)	0(0)	0(0)	0(0)	0(0)	0(0)	2(40)	0(0)	0(0)
Tetracycline	0(0)	0(0)	0(0)	0(0)	0(0)	0(0)	1(17)	2(33)	0(0)	1(14)	0(0)	0(0)	0(0)	0(0)	0(0)	2(40)	1(17)	0(0)	2(40)	2(33)	0(0)
Tigecycline	0(0)	0(0)	0(0)	0(0)	0(0)	0(0)	0(0)	0(0)	0(0)	0(0)	0(0)	0(0)	0(0)	0(0)	0(0)	0(0)	0(0)	0(0)	0(0)	0(0)	0(0)
Imipenem	0(0)	0(0)	0(0)	0(0)	0(0)	0(0)	0(0)	0(0)	0(0)	0(0)	0(0)	0(0)	0(0)	0(0)	0(0)	0(0)	0(0)	0(0)	0(0)	0(0)	1(17)
Meropenem	0(0)	0(0)	0(0)	0(0)	0(0)	0(0)	0(0)	0(0)	0(0)	0(0)	0(0)	0(0)	0(0)	0(0)	0(0)	0(0)	1(17)	0(0)	3(60)	1(17)	0(0)
NalidixicAcid	0(0)	3(100)	0(0)	0(0)	2(40)	1(17)	0(0)	1(17)	1(33)	6(86)	0(0)	1(20)	2(67)	0(0)	0(0)	3(60)	1(17)	0(0)	3(60)	1(17)	0(0)
Ciprofloxacin	0(0)	3(100)	0(0)	0(0)	1(20)	0(0)	0(0)	1(17)	0(0)	2(29)	0(0)	1(20)	0(0)	0(0)	0(0)	0(0)	1(17)	0(0)	2(40)	1(17)	0(0)
Co-trimoxazole	0(0)	1(33)	0(0)	0(0)	1(20)	0(0)	2(33)	2(33)	1(33)	3(43)	0(0)	1(20)	1(33)	0(0)	0(0)	3(60)	1(17)	0(0)	2(40)	2(33)	0(0)
Sulfamethizole	0(0)	1(33)	0(0)	0(0)	1(20)	0(0)	2(33)	1(17)	0(0)	3(43)	0(0)	1(20)	1(33)	0(0)	0(0)	2(40)	1(17)	0(0)	1(20)	2(33)	0(0)
Nitrofurantoin	0(0)	0(0)	0(0)	0(0)	1(20)	1(17)	0(0)	0(0)	0(0)	0(0)	0(0)	0(0)	0(0)	0(0)	0(0)	0(0)	0(0)	0(0)	0(0)	0(0)	0(0)
ESBL	0(0)	3(100)	0(0)	1(17)	1(20)	1(17)	3(50)	2(33)	0(0)	0(0)	0(0)	0(0)	1(33)	1(33)	0(0)	2(40)	2(33)	0(0)	0(0)	3(50)	0(0)

MDR	0(0)	3(100)	0(0)	0(0)	2(40)	1(17)	3(50)	4(67)	1(33)	3(43)	0(0)	1(20)	1(33)	0(0)	0(0)	2(40)	2(33)	0(0)	3(60)	2(33)	1(17)

**Antibiotic**	**Second year**																											
	**Summer**							**Rain**							**Autumn**							**Winter**						
	**Site 1 (N=6)**	**Site 2 (N=6)**	**Site 3 (N=7)**	**Site 4 (N=6)**	**Site 5 (N=6)**	**Site 6 (N=6)**	**Site 7 (N=6)**	**Site 1 (N=6)**	**Site 2 (N=4)**	**Site 3 (N=0)**	**Site 4 (N=6)**	**Site 5 (N=6)**	**Site 6 (N=6)**	**Site 7 (N=6)**	**Site 1 (N=1)**	**Site 2 (N=6)**	**Site 3 (N=6)**	**Site 4 (N=7)**	**Site 5 (N=6)**	**Site 6 (N=6)**	**Site 7 (N=1)**	**Site 1 (N=6)**	**Site 2 (N=6)**	**Site 3 (N=6)**	**Site 4 (N=5)**	**Site 5 (N=6)**	**Site 6 (N=6)**	**Site 7 (N=6)**
	**n(%)**	**n(%)**	**n(%)**	**n(%)**	**n(%)**	**n(%)**	**n(%)**	**n(%)**	**n(%)**	**n(%)**	**n(%)**	**n(%)**	**n(%)**	**n(%)**	**n(%)**	**n(%)**	**n(%)**	**n(%)**	**n(%)**	**n(%)**	**n(%)**	**n(%)**	**n(%)**	**n(%)**	**n(%)**	**n(%)**	**n(%)**	**n(%)**
Ampicillin	0(0)	0(0)	0(0)	0(0)	2(33)	0(0)	2(33)	2(33)	0(0)	0(0)	0(0)	1(17)	1(17)	1(17)	0(0)	0(0)	0(0)	1(14)	0(0)	1(17)	0(0)	3(50)	2(33)	4(67)	5(100)	1(17)	0(0)	4(67)
Cefotaxime	0(0)	0(0)	0(0)	0(0)	1(17)	0(0)	2(33)	2(33)	0(0)	0(0)	0(0)	1(17)	1(17)	0(0)	0(0)	0(0)	0(0)	1(14)	0(0)	0(0)	0(0)	0(0)	1(17)	2(33)	5(100)	0(0)	0(0)	4(67)
Ceftazidime	0(0)	0(0)	0(0)	0(0)	0(0)	0(0)	0(0)	2(33)	0(0)	0(0)	0(0)	1(17)	1(17)	0(0)	0(0)	0(0)	0(0)	0(0)	0(0)	0(0)	0(0)	0(0)	0(0)	3(50)	5(100)	0(0)	0(0)	4(67)
Cefepime	0(0)	0(0)	0(0)	0(0)	0(0)	0(0)	0(0)	1(17)	0(0)	0(0)	0(0)	1(17)	0(0)	0(0)	0(0)	0(0)	0(0)	0(0)	0(0)	0(0)	0(0)	0(0)	0(0)	2(33)	2(40)	0(0)	0(0)	4(67)
Amikacin	0(0)	0(0)	0(0)	0(0)	0(0)	0(0)	0(0)	0(0)	0(0)	0(0)	0(0)	0(0)	0(0)	0(0)	0(0)	0(0)	0(0)	0(0)	0(0)	0(0)	0(0)	0(0)	0(0)	0(0)	0(0)	0(0)	0(0)	0(0)
Gentamicin	0(0)	0(0)	0(0)	0(0)	0(0)	0(0)	0(0)	0(0)	0(0)	0(0)	0(0)	0(0)	0(0)	0(0)	0(0)	0(0)	0(0)	0(0)	0(0)	0(0)	0(0)	0(0)	0(0)	0(0)	0(0)	0(0)	0(0)	0(0)
Tetracycline	0(0)	0(0)	1(14)	0(0)	0(0)	0(0)	2(33)	1(17)	0(0)	0(0)	0(0)	1(17)	0(0)	1(17)	0(0)	0(0)	1(17)	0(0)	1(17)	1(17)	0(0)	0(0)	0(0)	1(17)	3(60)	0(0)	0(0)	1(17)
Tigecycline	0(0)	0(0)	0(0)	0(0)	0(0)	0(0)	0(0)	0(0)	0(0)	0(0)	0(0)	0(0)	0(0)	0(0)	0(0)	0(0)	0(0)	0(0)	0(0)	0(0)	0(0)	0(0)	0(0)	0(0)	0(0)	0(0)	0(0)	0(0)
Imipenem	0(0)	0(0)	0(0)	0(0)	0(0)	0(0)	0(0)	0(0)	0(0)	0(0)	0(0)	0(0)	0(0)	0(0)	1(100)	0(0)	1(17)	0(0)	0(0)	0(0)	0(0)	0(0)	0(0)	0(0)	0(0)	0(0)	0(0)	0(0)
Meropenem	0(0)	0(0)	0(0)	0(0)	0(0)	0(0)	0(0)	0(0)	0(0)	0(0)	0(0)	0(0)	0(0)	0(0)	0(0)	0(0)	0(0)	0(0)	0(0)	0(0)	0(0)	0(0)	2(33)	0(0)	3(60)	0(0)	0(0)	3(50)
NalidixicAcid	0(0)	1(17)	0(0)	0(0)	0(0)	0(0)	0(0)	1(17)	1(25)	0(0)	1(17)	3(50)	0(0)	0(0)	0(0)	0(0)	2(33)	2(29)	0(0)	0(0)	1(100)	0(0)	2(33)	2(33)	4(80)	0(0)	1(17)	5(83)
Ciprofloxacin	0(0)	1(17)	0(0)	0(0)	0(0)	0(0)	0(0)	1(17)	1(25)	0(0)	1(17)	2(33)	0(0)	0(0)	0(0)	0(0)	0(0)	0(0)	0(0)	0(0)	0(0)	0(0)	0(0)	1(17)	4(80)	0(0)	0(0)	1(17)
Co-trimoxazole	0(0)	0(0)	1(14)	0(0)	3(50)	0(0)	2(33)	1(17)	0(0)	0(0)	0(0)	1(17)	0(0)	0(0)	0(0)	0(0)	1(17)	1(14)	1(17)	0(0)	0(0)	2(33)	2(33)	2(33)	4(80)	0(0)	0(0)	1(17)
Sulfamethizole	0(0)	0(0)	1(14)	0(0)	0(0)	0(0)	1(17)	1(17)	0(0)	0(0)	0(0)	1(17)	0(0)	0(0)	1(100)	0(0)	1(17)	1(14)	1(17)	0(0)	1(100)	2(33)	2(33)	1(17)	4(80)	0(0)	0(0)	0(0)

Nitrofurantoin	0(0)	0(0)	0(0)	0(0)	0(0)	0(0)	0(0)	0(0)	0(0)	0(0)	0(0)	0(0)	0(0)	0(0)	5(83)	0(0)	0(0)	0(0)	0(0)	0(0)	5(83)	0(0)	0(0)	1(17)	0(0)	0(0)	0(0)	0(0)
ESBL	0(0)	0(0)	0(0)	0(0)	1(17)	0(0)	1(17)	2(33)	0(0)	0(0)	0(0)	1(17)	1(17)	0(0)	0(0)	0(0)	0(0)	1(14)	0(0)	0(0)	0(0)	0(0)	1(17)	5(83)	3(60)	0(0)	1(17)	4(67)
MDR	0(0)	0(0)	1(15)	0(0)	1(17)	0(0)	2(33)	1(17)	0(0)	0(0)	0(0)	1(17)	0(0)	0(0)	1(100)	0(0)	1(17)	1(14)	1(17)	1(17)	1(100)	2(33)	2(33)	3(50)	4(80)	0(0)	1(17)	5(83)

**Antibiotic**	**Third year**																											
	**Summer**							**Rain**							**Autumn**							**Winter**						
	**Site 1 (N=6)**	**Site 2 (N=0)**	**Site 3 (N=6)**	**Site 4 (N=6)**	**Site 5 (N=6)**	**Site 6 (N=6)**	**Site 7 (N=1)**	**Site 1 (N=6)**	**Site 2 (N=6)**	**Site 3 (N=6)**	**Site 4 (N=6)**	**Site 5 (N=6)**	**Site 6 (N=6)**	**Site 7 (N=6)**	**Site 1 (N=6)**	**Site 2 (N=6)**	**Site 3 (N=6)**	**Site 4 (N=6)**	**Site 5 (N=6)**	**Site 6 (N=6)**	**Site 7 (N=6)**	**Site 1 (N=6)**	**Site 2 (N=6)**	**Site 3 (N=6)**	**Site 4 (N=0)**	**Site 5 (N=6)**	**Site 6 (N=6)**	**Site 7 (N=0)**
	**n(%)**	**n(%)**	**n(%)**	**n(%)**	**n(%)**	**n(%)**	**n(%)**	**n(%)**	**n(%)**	**n(%)**	**n(%)**	**n(%)**	**n(%)**	**n(%)**	**n(%)**	**n(%)**	**n(%)**	**n(%)**	**n(%)**	**n(%)**	**n(%)**	**n(%)**	**n(%)**	**n(%)**	**n(%)**	**n(%)**	**n(%)**	**n(%)**
Ampicillin	0(0)	0(0)	0(0)	0(0)	0(0)	0(0)	0(0)	1(17)	2(33)	0(0)	2(33)	6(100)	4(67)	6(100)	6(100)	5(83)	0(0)	3(50)	6(100)	1(17)	6(100)	0(0)	4(67)	0(0)	0(0)	0(0)	2(33)	0(0)
Cefotaxime	0(0)	0(0)	0(0)	0(0)	0(0)	0(0)	0(0)	1(17)	0(0)	0(0)	1(17)	0(0)	3(50)	2(33)	0(0)	1(17)	0(0)	3(50)	0(0)	1(17)	0(0)	0(0)	3(50)	0(0)	0(0)	0(0)	2(33)	0(0)
Ceftazidime	0(0)	0(0)	0(0)	0(0)	0(0)	0(0)	0(0)	1(17)	0(0)	0(0)	1(17)	0(0)	3(50)	2(33)	1(17)	0(0)	0(0)	2(33)	0(0)	1(17)	0(0)	0(0)	3(50)	0(0)	0(0)	0(0)	2(33)	0(0)
Cefepime	0(0)	0(0)	0(0)	0(0)	0(0)	0(0)	0(0)	1(17)	0(0)	0(0)	1(17)	0(0)	3(50)	2(33)	0(0)	0(0)	0(0)	1(17)	0(0)	1(17)	0(0)	0(0)	3(50)	0(0)	0(0)	0(0)	2(33)	0(0)
Amikacin	0(0)	0(0)	0(0)	0(0)	0(0)	0(0)	0(0)	0(0)	0(0)	0(0)	0(0)	0(0)	0(0)	0(0)	0(0)	0(0)	0(0)	0(0)	0(0)	0(0)	0(0)	0(0)	0(0)	0(0)	6(100)	0(0)	0(0)	6(100)
Gentamicin	0(0)	0(0)	0(0)	0(0)	0(0)	0(0)	0(0)	0(0)	0(0)	0(0)	0(0)	0(0)	0(0)	0(0)	0(0)	0(0)	0(0)	0(0)	0(0)	0(0)	0(0)	0(0)	0(0)	0(0)	0(0)	0(0)	0(0)	0(0)
Tetracycline	0(0)	0(0)	0(0)	0(0)	0(0)	0(0)	0(0)	0(0)	0(0)	0(0)	0(0)	0(0)	0(0)	0(0)	1(17)	0(0)	0(0)	2(33)	0(0)	0(0)	0(0)	0(0)	4(67)	0(0)	0(0)	1(17)	0(0)	0(0)
Tigecycline	0(0)	0(0)	0(0)	0(0)	0(0)	0(0)	0(0)	0(0)	0(0)	0(0)	0(0)	0(0)	0(0)	0(0)	0(0)	0(0)	0(0)	0(0)	0(0)	0(0)	0(0)	0(0)	0(0)	0(0)	0(0)	0(0)	0(0)	0(0)
Imipenem	0(0)	0(0)	0(0)	0(0)	0(0)	0(0)	0(0)	0(0)	0(0)	0(0)	0(0)	0(0)	1(17)	0(0)	0(0)	0(0)	6(100)	1(17)	0(0)	0(0)	1(17)	0(0)	0(0)	0(0)	0(0)	0(0)	0(0)	0(0)
Meropenem	0(0)	0(0)	0(0)	0(0)	0(0)	0(0)	0(0)	1(17)	0(0)	0(0)	0(0)	0(0)	0(0)	0(0)	2(33)	0(0)	0(0)	2(33)	0(0)	0(0)	0(0)	0(0)	1(17)	0(0)	0(0)	0(0)	0(0)	0(0)
NalidixicAcid	0(0)	0(0)	0(0)	0(0)	0(0)	0(0)	0(0)	2(33)	0(0)	0(0)	0(0)	0(0)	4(67)	2(33)	2(33)	2(33)	0(0)	3(50)	0(0)	0(0)	1(17)	0(0)	6(100)	0(0)	0(0)	1(17)	0(0)	0(0)
Ciprofloxacin	0(0)	0(0)	0(0)	0(0)	0(0)	0(0)	0(0)	1(17)	0(0)	0(0)	0(0)	0(0)	2(33)	0(0)	2(33)	0(0)	0(0)	2(33)	0(0)	0(0)	0(0)	0(0)	1(17)	0(0)	0(0)	0(0)	0(0)	0(0)
Co-trimoxazole	0(0)	0(0)	0(0)	0(0)	0(0)	0(0)	0(0)	1(17)	1(17)	0(0)	0(0)	0(0)	2(33)	0(0)	1(17)	0(0)	0(0)	3(50)	1(17)	0(0)	1(17)	0(0)	1(17)	0(0)	0(0)	0(0)	2(33)	0(0)

Sulfamethizole	0(0)	0(0)	0(0)	0(0)	0(0)	0(0)	0(0)	1(17)	1(17)	0(0)	0(0)	0(0)	2(33)	0(0)	1(17)	0(0)	0(0)	3(50)	1(17)	0(0)	1(17)	0(0)	1(17)	0(0)	0(0)	0(0)	2(33)	0(0)
Nitrofurantoin	0(0)	0(0)	0(0)	0(0)	0(0)	0(0)	0(0)	0(0)	0(0)	0(0)	0(0)	0(0)	0(0)	1(17)	0(0)	0(0)	0(0)	0(0)	0(0)	0(0)	0(0)	0(0)	0(0)	0(0)	0(0)	0(0)	0(0)	0(0)
ESBL	0(0)	0(0)	0(0)	0(0)	0(0)	0(0)	0(0)	1(17)	0(0)	0(0)	1(17)	0(0)	3(50)	2(33)	0(0)	0(0)	0(0)	2(33)	0(0)	1(17)	0(0)	0(0)	3(50)	0(0)	0(0)	0(0)	1(17)	0(0)
MDR	0(0)	0(0)	0(0)	0(0)	0(0)	0(0)	0(0)	1(17)	1(17)	0(0)	0(0)	0(0)	3(50)	0(0)	2(33)	0(0)	0(0)	3(50)	1(17)	1(17)	2(33)	0(0)	4(67)	0(0)	0(0)	0(0)	2(33)	0(0)

**Antibiotic**	**P-value***
Ampicillin	<0.0001
Cefotaxime	0.034
Ceftazidime	0.009
Cefepime	0.46
Amikacin	-
Gentamicin	-
Tetracycline	0.27
Tigecycline	-
Imipenem	0.26
Meropenem	0.02
Nalidixic Acid	0.15
Ciprofloxacin	0.07
Co-trimoxazole	0.31
Sulfamethizole	0.51
Nitrofurantoin	0.02
ESBL	<0.0001
MDR	<.00001

Abbreviations: ESBL: Extended Spectrum Beta-Lactamase, MDR: multidrug resistance, N = total number of *E. coli* isolates, n = number of resistant *E coli* isolates.

Note: *p-value are extracted from Analysis of Variance (ANOVA).

Table S7. Seasonal pairwise comparison of antibiotic resistant *E. coli* in waters of the Kshipra river in India over a 3-year period.

**River water samples**								
**Antibiotics**								
**Comparison**	Ampicillin	Cefepime	Amikacin	Tetracycline	Meropenem	Nalidixic Acid	Corimoxazole	Sulfamethizole
First Year								
Autumn -Rain	0.96008	0.99	0.99538	0.99	0.83473	0.99999	0.99	0.99
Autumn -Summer	0.02589	0.6596	0.00849	0.95054	0.99479	0.9999	0.18202	0.62347
Autumn -Winter	0.99	0.90704	<0.0001	0.8388	<0.0001	0.86741	0.99	0.99
Rain -Summer	0.82353	0.88761	<0.0001	0.99074	0.04456	0.99	0.4307	0.26889
Rain -Winter	0.99987	0.56969	<0.0001	0.54466	<0.0001	0.20936	0.9998	0.99
Summer -Winter	0.14785	0.00213	<0.0001	0.01217	<0.0001	0.16285	0.02464	0.67985
Second year								
Autumn -Rain	0.39416	0.99	<0.0001	0.99	0.99	0.98782	0.63444	0.99935
Autumn -Summer	0.99	0.99434	<0.0001	0.78695	0.98899	0.9927	0.99	0.25586
Autumn -Winter	0.99	0.08086	0.9942	0.22955	0.01597	0.98748	0.99997	0.69428
Rain -Summer	0.75714	0.7558	0.99	0.34582	0.9769	0.99	0.70573	0.92925
Rain -Winter	0.97612	0.25585	0.21739	0.051	0.00922	0.99	0.99979	0.99769
Summer -Winter	0.99	0.00053	0.10614	0.99986	<0.0001	0.99	0.99999	0.99
Third Year								
Autumn -Rain	0.99795	0.99	0.99	0.98865	0.0407	0.99	0.99	0.99
Autumn -Summer	<0.0001	0.03381	0.99999	0.03818	0.00095	0.99	0.99	0.13142
Autumn -Winter	<0.0001	0.31961	0.99977	0.05214	0.00648	0.99994	0.92492	0.99
Rain -Summer	<0.0001	0.04278	0.99	0.76055	0.99936	0.99	0.99	0.53639
Rain -Winter	<0.0001	0.202	0.99	0.84545	0.99	0.99796	0.50139	0.99
Summer -Winter	0.99	<0.0001	0.99	0.99	0.99	0.99	0.70074	0.32455

Note: p-value adjusted with Tukey method.

Table S8. Seasonal pairwise comparison of antibiotic resistant *E. coli* isolated from sediments of the Kshipra river in India over a 3-year period.

River sediment samples					
**Comparison**	Ampicillin	Cefotaxime	Ceftazidime	Meropenem	Nitrofurantoin
**First year**					
Autumn – Rain	0.99	0.9982	0.99	0.83978	0.99
Autumn – Winter	0.99828	0.94097	0.98354	<0.0001	0.86443
Rain – Winter	0.96024	0.99	0.99188	<0.0001	0.99849
**Second year**					
Autumn – Rain	0.94499	0.99	0.99	0.96374	<0.0001
Autumn – Summer	0.99995	0.99	0.99	0.99	<0.0001
Autumn – Winter	<0.0001	0.0147	<0.0001	0.00046	<0.0001
Rain – Summer	0.99998	0.99	0.99974	0.96835	0.99
Rain – Winter	0.05185	0.0262	<0.0001	<0.0001	0.99
Summer – Winter	0.00054	0.00061	<0.0001	<0.0001	0.99
**Third year**					
Autumn – Rain	0.99999	0.9917	0.99998	0.98663	0.99999
Autumn – Summer	<0.0001	0.99893	0.98343	0.28637	0.99999
Autumn – Winter	<0.0001	0.99996	0.99	0.10641	0.99
Rain – Summer	<0.0001	0.45377	0.57557	0.99442	0.99

Rain – Winter	0.00088	0.99	0.99	0.92798	0.99
Summer – Winter	0.96272	0.84506	0.93842	0.99	0.99

Note: p-value adjusted with Tukey method.

Table S9. Average values (standard deviation) of water quality parameters of the Kshipra river in India in various seasons and at various sites over a 3-year period.

**Season**	**Year**	**Site**	**Abtemt. (°C)**	**Wtemp. (°C)**	**PH**	**Cond. (µS/cm)**	**TDS (mg/L)**	**DO (mg/L)**	**Turb. (NTU)**	**TSS (mg/L)**	**BOD (mg/L)**	**COD (mg/L)**	**Free CO_2 (mg/L)**	**CO_3Alk (mg/L)**
Autumn	First	1	31(0)	29(0)	8,7(0)	1105,33(22)	586,67(5)	6,27(0,20)	21,1(2,10)	60(3)	13,34(2)	37,34(8)	20(0)	12,67(8)
Rain	First	1	30(0)	28(0)	8,4(0)	1311,47(17)	800(10,44)	4,4(0)	NA	70(4,18)	28(4,18)	80(8,36)	20(0)	2,5(0,52)
Summer	First	1	39(0)	33(0)	8,55(0,05)	1200(10,44)	705(5,22)	8(0,42)	81,5(0,52)	91,5(5,74)	18(2,09)	48(8,36)	20(0)	4,5(0,52)
Winter	First	1	18(0)	19(0)	8,7(0)	1362(8,36)	890(0)	5,2(0)	15,96(0,51)	75,5(7,83)	20(0)	56(8,36)	20(0)	16(0)
Autumn	Second	1	31(0)	29(0)	8,4(0)	1453(17,76)	885(5,22)	4,8(0)	23,2(0,31)	59(3,13)	11,6(0,42)	38(2,09)	20(0)	10,5(3,66)
Rain	Second	1	25(0)	26(0)	7,55(0,05)	415(4,18)	253,5(2,61)	6(0)	605,5(9,92)	654,5(12,01)	6,4(0)	32(4,18)	15,5(1,57)	0(0)
Summer	Second	1	33(0)	29(0)	8,95(0,05)	974,5(19,32)	604,5(1,57)	4,4(0)	48,7(3,66)	208,25(6,53)	48(16,71)	172(29,25)	20(0)	8,5(0,52)
Winter	Second	1	20(0)	20(0)	8,9(0)	1170(64,76)	835(5,22)	10,6(0,21)	24,85(0,47)	20,15(0,37)	25,6(2,09)	54(2,09)	20(0)	10(1,04)
Autumn	Third	1	25(0)	22(0)	6,4(0)	902,5(19,32)	550(10,44)	7,4(0,63)	21,1(2,51)	38(2,09)	14,6(1,04)	42(2,09)	12,5(0,52)	0(0)
Rain	Third	1	25(0)	26(0)	8,55(0,05)	258(2,09)	170(0)	5,6(0)	108,15(1,72)	82,25(2,35)	4,6(1,04)	22(2,09)	19(0)	0(0)
Summer	Third	1	32(0)	28(0)	8,6(0)	376,5(13,06)	235(5,22)	6,8(0,84)	15,5(1,36)	43(9,40)	7,2(0,42)	20(4,18)	20(0)	4,5(0,52)
Winter	Third	1	17(0)	19(0)	8,1(0)	882(104,45)	600(10,44)	5,8(0,21)	2,08(0,15)	7,5(1,57)	31,2(5,01)	68(4,18)	15,5(4,70)	7(7,31)
Autumn	First	2	29(0)	30(0)	8,2(0)	1274(13,50)	803,34(10)	4,94(0,20)	6,08(0,05)	27,67(3,50)	29,33(4)	80(0)	3,67(0,50)	0(0)
Rain	First	2	30(0)	29(0)	8,2(0)	1459,01(0)	890(0)	4,2(0)	NA	93(7,31)	52(4,18)	160(25,07)	2,5(0,52)	0(0)
Summer	First	2	40(0)	34(0)	7,66(0,05)	1431,67(10,30)	861,67(10,30)	0,97(0,21)	102,84(1,03)	130,67(4,12)	63(6,18)	201,34(8,24)	5,58(0,51)	0(0)
Winter	First	2	23(0)	19(0)	8,45(0,05)	1235(5,22)	855(5,22)	5,2(0)	8,065(1,32)	42,5(10,97)	36(4,18)	96(16,71)	14,5(3,66)	0(0)
Autumn	Second	2	32(0)	30(0)	8,4(0)	1447,69(2,59)	875,38(5,19)	6(0)	22,87(0,36)	36,15(8,82)	12,36(0,42)	46,15(2,08)	18(0)	0(0)
Rain	Second	2	25(0)	25(0)	7,6(0)	336,5(2,61)	205,5(1,57)	4,9(0,10)	465,5(63,19)	624(6,27)	7,2(0)	46(18,80)	15,5(0,52)	0(0)

Summer	Second	2	34(0)	31(0)	8,3(0)	1122,5(7,83)	685(3,13)	5(0,21)	82,3(3,86)	216(3,13)	72(0)	184(8,36)	6,5(0,52)	0(0)
Winter	Second	2	21(0)	19(0)	8,5(0)	1315,5(55,88)	930(0)	14,4(0,42)	38,9(1,78)	54,6(2,51)	40,6(1,04)	66(2,09)	20(0)	15(1,04)
Autumn	Third	2	25(0)	23(0)	7,4(0)	1252(2,09)	775(5,22)	7(0,21)	18,05(0,37)	55(2,09)	35,6(4,18)	56(4,18)	4(0)	0(0)
Rain	Third	2	25(0)	26(0)	8,1(0)	732,5(30,81)	465(5,22)	5,6(0,42)	82,85(2,56)	112,25(3,39)	6,6(1,04)	30(2,09)	19(0)	0(0)
Summer	Third	2	33(0)	29(0)	8,5(0)	1068(42,82)	635(5,22)	6,2(0,21)	26,15(1,72)	66,75(6,53)	10,8(0,84)	24(4,18)	20(0)	2,5(0,52)
Winter	Third	2	18(0)	19(0)	8,2(0)	1106,5(18,28)	685(5,22)	4,6(1,46)	0,4(0,04)	3,25(0,78)	21,8(2,72)	38(2,09)	20(0)	15(1,04)
Autumn	First	3	35(0)	31(0)	8,95(0,05)	1149,5(6,79)	625(5,22)	10,4(0)	21,3(0,42)	88(8,36)	56(0)	168(8,36)	20(0)	4(0)
Rain	First	3	30(0)	29(0)	8,2(0)	1459,02(0)	890(0)	4,8(0)	NA	61,5(1,57)	40(0)	108(4,18)	18,5(0,52)	0(0)
Summer	First	3	41(0)	34(0)	8,35(0,05)	1345(5,22)	840(10,44)	5,8(0,21)	90(4,18)	81,5(7,83)	40(0)	124(4,18)	20(0)	4,5(0,52)
Winter	First	3	25(0)	20(0)	8,4(0)	1372,5(7,83)	870(0)	4,8(0)	16,62(1,90)	63,5(16,19)	58(2,09)	160(16,71)	11(0)	0(0)
Autumn	Second	3	34(0)	30(0)	8,3(0)	1417,5(2,61)	860(0)	5(0,21)	22,3(0,42)	54,75(5,57)	11,6(0,42)	42(2,09)	18(0)	0(0)
Rain	Second	3	25(0)	25(0)	7,7(0)	469(13,58)	286(8,36)	6(0)	567(15,67)	610(12,53)	5,6(0)	22(2,09)	11(0)	0(0)
Summer	Second	3	36(0)	30(0)	8,1(0)	1133,5(14,10)	687,5(2,61)	4,8(0,42)	69,15(3,29)	151,75(3,39)	60(4,18)	148(4,18)	4,5(1,57)	0(0)
Winter	Second	3	25(0)	20(0)	9,1(0)	1163(29,25)	815(5,22)	22,6(1,04)	31,55(0,26)	49,75(2,35)	27,6(0)	66(6,27)	20(0)	12,5(0,52)
Autumn	Third	3	28(0)	24(0)	7,6(0)	1108(21,93)	650(0)	6,4(0)	39,65(0,89)	116,75(6,01)	38,6(9,40)	78(6,27)	5(0)	0(0)
Rain	Third	3	25(0)	26(0)	8,1(0)	574,5(2,61)	340(0)	6,2(0,21)	74,85(3,71)	109,25(2,87)	6,6(5,22)	18(6,27)	3,5(0,52)	0(0)
Summer	Third	3	33(0)	28(0)	8,5(0)	279,5(1,57)	175(5,22)	7(0,63)	20,85(0,68)	46(2,61)	7,6(0,84)	46(6,27)	20(0)	9(6,27)
Winter	Third	3	24(0)	19(0)	8,1(0)	1048(25,07)	640(0)	9,4(6,06)	1,385(0,20)	4(0,52)	20,6(0,21)	44(4,18)	11(0)	0(0)
Autumn	First	4	35(0)	32(0)	8,3(0)	1301(5,22)	770(0)	4(0)	12,58(1,94)	58(8,36)	36(4,18)	112(16,71)	14,5(3,66)	0(0)
Rain	First	4	31(0)	28(0)	8,3(0)	1393,44(0)	850(0)	4,8(0,42)	NA	72(6,27)	28(4,18)	84(4,18)	20(0)	2(0)
Summer	First	4	39(0)	32(0)	8,35(0,05)	1325(5,22)	825(5,22)	7,4(0,21)	69(3,13)	68(2,09)	26(2,09)	74(6,27)	20(0)	0(0)
Winter	First	4	26(0)	19(0)	9,2(0)	1496(6,27)	935(5,22)	9,4(0,21)	140,495(20,81)	195(33,42)	30(2,09)	80(0)	20(0)	7,5(1,57)
Autumn	Second	4	34(0)	32(0)	8,45(0,05)	1374(14,62)	845(5,22)	7,8(0,21)	34(1,04)	62,75(4,96)	12,8(0,84)	42(6,27)	20(0)	2(0)
Rain	Second	4	24(0)	25(0)	7,65(0,05)	308,5(6,79)	188(4,18)	5,4(0,21)	547(11,49)	654,5(12,01)	6,8(0,42)	34(6,27)	10,5(0,52)	0(0)
Summer	Second	4	39(0)	32(0)	8,280)	1098(43,87)	678,5(0,52)	3,6(0)	75,5(6,79)	105,5(3,66)	40(0)	108(4,18)	20(0)	15(1,04)
Winter	Second	4	27(0)	20(0)	8,7(0)	1144,5(15,14)	850(10,44)	6(0,42)	19,9(0,42)	17,05(1,51)	13,6(2,09)	34(2,09)	20(0)	16(2,09)
Autumn	Third	4	29(0)	23(0)	7,5(0)	1088,5(14,10)	680(0)	6(0,42)	20,4(1,15)	37,5(5,22)	20,6(3,13)	62(6,27)	4,5(0,52)	0(0)
Rain	Third	4	25(0)	27(0)	8,1(0)	508(19,84)	305(5,22)	6,2(0,21)	57,95(4,86)	103,5(1,57)	4,6(3,13)	26(6,27)	10,5(8,88)	0(0)
Summer	Third	4	35(0)	29(0)	8,1(0)	377,5(12,01)	240(0)	5,6(0,42)	15,6(1,15)	38,25(5,48)	8(0,42)	22(2,09)	2(0)	0(0)
Winter	Third	4	23(0)	20(0)	8,2(0,10)	1010(26,11)	620(10,44)	8,2(6,89)	11,345(3,61)	67,25(21,15)	26,2(1,88)	52(8,36)	20(0)	2,5(0,52)

Autumn	First	5	27(0)	28(0)	8,5(0)	1177,5(84,08)	805(5,22)	4,4(0,21)	18,1(1,55)	73,5(6,79)	32(0)	88(8,36)	18(0)	0(0)
Rain	First	5	28(0)	29(0)	8,4(0)	1360,66(0)	830(0)	4,6(0,21)	NA	86,5(3,66)	36(4,18)	104(8,36)	20(0)	17(1,04)
Summer	First	5	39(0)	33,5(0,52)	8,45(0,05)	1325(5,22)	830(20,89)	8,6(0,21)	73(1,04)	77(5,22)	30(2,09)	84(4,18)	20(0)	5(1,04)
Winter	First	5	16(0)	19(0)	8,4(0)	1440(20,89)	915(5,22)	4,6(0,21)	21,6(0,10)	56,5(5,74)	22(2,09)	64(0)	18(0)	0(0)
Autumn	Second	5	33(0)	30(0)	8,3(0)	1330(20,89)	820(09	2,6(0,21)	15(0,21)	57,75(11,75)	8,4(0,42)	26(2,09)	18(0)	0(0)
Rain	Second	5	26(0)	25(0)	7,78(0)	321(4,18)	195,5(2,61)	6(0)	537(47)	575(13,58)	9,2(0,42)	34(2,09)	8,5(0,52)	0(0)
Summer	Second	5	35(0)	31(0)	8(0)	1117,5(5,74)	679(4,18)	1(0,21)	54(2,09)	68(9,40)	68(4,18)	152(8,36)	10,5(8,88)	0(0)
Winter	Second	5	22(0)	19(0)	8,9(0)	956,5(31,86)	715(5,22)	8,4(0)	21,05(3,71)	42,35(4,02)	19,6(09	42(2,09)	20(0)	10(0)
Autumn	Third	5	23(0)	21(0)	7,5(0)	1097(8,36)	705(5,229	4,2(1,46)	24,35(1,51)	65,5(15,67)	30,8(4,18)	54(6,27)	2,5(0,52)	0(0)
Rain	Third	5	23(0)	24(0)	8,05(0,05)	421,5(5,74)	265(5,22)	5,8(0,21)	160,45(20,84)	185,5(27,68)	6,6(1,04)	26(2,09)	18(0)	0(0)
Summer	Third	5	32(0)	30(0)	8,05(0,05)	575,5(112,28)	375(57,45)	5,8(0,21)	20,7(1,36)	51,5(3,13)	8,4(0,42)	26(6,27)	18,5(0,52)	0(0)
Winter	Third	5	17(0)	17(0)	8(0)	999(17,76)	625(5,22)	3,2(0,42)	7,35(0,14)	71,5(2,61)	41,2(6,27)	66(6,27)	20(0)	17(1,04)
Autumn	First	6	30(0)	28(0)	8,45(0,05)	1192(16,71)	800(0)	3,6(0)	18,52(0,17)	123,5(6,79)	68(4,18)	192(16,71)	11(0)	0(0)
Rain	First	6	28(0)	29(0)	8,35(0,05)	1204,91(8,56)	735(5,22)	3,4(0,21)	NA	135(20,89)	68(4,18)	196(4,18)	20(0)	15(1,04)
Summer	First	6	39(0)	33(0)	8,35(0,05)	1350(41,78)	835(15,67)	7,4(0,21)	85(1,04)	80,5(1,57)	38(2,09)	100(4,18)	20(0)	3(1,04)
Winter	First	6	20(0)	21(0)	8,5(0)	1420(15,67)	920(0)	4,6(0,21)	41,15(3,92)	74(3,13)	66(6,27)	200(8,36)	19(0)	0(0)
Autumn	Second	6	33(0)	29(0)	8,1(0)	1344,5(10,97)	820(10,44)	2,6(0,21)	22,3(0,94)	64,75(2,87)	12,4(0,42)	34(2,09)	13(2,09)	0(0)
Rain	Second	6	28(0)	25(0)	7,6(0)	363,5(2,61)	221,5(1,57)	5,8(0,21)	490,5(22,46)	591(28,20)	12(0,84)	42(6,27)	10(0)	0(0)
Summer	Second	6	36(0)	31(0)	7,8(0)	1151(5,22)	685(0)	0,8(0)	83(5,22)	94(12,53)	76(12,53)	156(20,89)	10,5(8,88)	0(0)
Winter	Second	6	25(0)	20(0)	8,6(0)	986,5(25,59)	745(5,22)	7(0,21)	27(0,21)	84,5(4,18)	36,6(1,04)	68(4,18)	20(0)	5,5(0,52)
Autumn	Third	6	23(0)	22(0)	7,4(0)	1113,5(10,97)	700(0)	4,2(1,88)	17,65(0,37)	70(14,62)	21,8(1,04)	44(4,18)	18(0)	0(0)
Rain	Third	6	23(0)	24(0)	8,05(0,05)	403,5(0,52)	265(5,22)	6,2(0,63)	426,5(24,54)	367,5(18,28)	9,6(2,09)	34(2,09)	18,5(0,52)	0(0)
Summer	Third	6	33(0)	30(0)	8,2(0)	694(69,98)	470(10,44)	6,2(0,21)	25,7(1,15)	72,25(9,66)	9,6(0,42)	28(4,18)	20(0)	15(1,04)
Winter	Third	6	17(0)	18(0)	8,05(0,05)	1032,5(4,70)	615(5,22)	4,6(1,46)	10,34(1,72)	26,75(3,92)	33,2(2,09)	54(2,09)	20(0)	16,5(0,52)
Autumn	First	7	32(0)	28(0)	8,4(0)	1192,5(28,72)	785(5,22)	5(0)	14,79(3,03)	70(5,22)	18(20,9)	56(8,36)	11(0)	0(0)
Rain	First	7	27(0)	29(0)	8,45(0,05)	1393,44(0)	850(0)	6(0)	NA	96(4,18)	20(4,18)	52(12,53)	20(0)	15(1,04)
Summer	First	7	38(0)	31(0)	8,3(0)	1325(5,22)	820(0)	7,6(0,21)	31(3,13)	43(3,13)	14(2,09)	36(4,18)	20(0)	4,5(0,52)
Winter	First	7	23(0)	19(0)	8,65(0,05)	1305(10,44)	840(0)	5(0,21)	36,55(2,87)	39(6,27)	18(6,27)	34,5(14,10)	20(0)	17(1,04)
Autumn	Second	7	32(0)	29(0)	8,3(0)	1306(16,71)	790(10,44)	3,8(0,21)	17,05(0,57)	45,25(1,31)	8,8(1,67)	30(2,09)	18,5(0,52)	0(0)
Rain	Second	7	25(0)	25(0)	7,6(0)	460(5,22)	281(3,13)	5,8(0,21)	640,5(14,10)	681(15,67)	10(2,09)	34(6,27)	11(0)	0(0)

Summer	Second	7	37(0)	32(0)	8,3(0)	1131(5,22)	678,5(0,52)	4,6(0,21)	29(7,31)	65(3,13)	44(4,18)	132(4,18)	20(0)	17(1,04)
Winter	Second	7	26(0)	21(0)	8,4(0)	967(41,78)	750(0)	5(0,21)	11,2(0,31)	52(4,18)	16,6(1,04)	48(8,36)	20(0)	15(1,04)
Autumn	Third	7	25(0)	22(0)	7,7(0)	1102(13,58)	715(5,22)	5,6(0)	13,2(0,84)	65,25(20,63)	18,8(4,18)	54(10,44)	14,5(3,66)	0(0)
Rain	Third	7	23(0)	24(0)	8,05(0,05)	507,5(15,14)	310(0)	7,4(1,04)	128,15(25,33)	99,5(21,41)	6,6(1,04)	26(2,09)	10,5(8,88)	0(0)
Summer	Third	7	34(0)	31(0)	8,3(0)	828(72,07)	535(26,11)	6(0)	11,5(0,31)	27,25(4,44)	7,4(0,63)	28(4,18)	20(0)	16(0)
Winter	Third	7	18(0)	16(0)	8(0)	950(56,40)	600(0)	6(0)	11,745(2,62)	116(22,98)	37,2(2,09)	56(4,18)	20(0)	11,5(6,79)
**P-value**			**<0.0001**	**<0.0001**	**<0.0001**	**<0.0001**	**<0.0001**	**<0.0001**	**<0.0001**	**<0.0001**	**<0.0001**	**<0.0001**	**<0.0001**	**<0.0001**

Cont. Table S9. Average values (standard deviation) of water quality parameters of the Kshipra river in India in various seasons and at various sites over a 3-year period.

HCO3 Alk (mg/L)	Talk. (mg/L)	Cl (mg/L)	TH (mg/L)	CaH (mg/L)	MgH (mg/L)	NO3-N (mg/L)	TP (mg/L)	OrthoP (mg/L)	OrgP (mg/L)	CFU/100 (TCC)	CFU/100ml (TEC)
228(6)	236,67(7)	122,211(2,50)	278(3)	158,9(1,05)	119,1(1,95)	3,5(0,15)	2,27(0,02)	1,834(0,02)	0,4367(0,01)	91(48)	6,67(0,50)
218(6,27)	229(7,31)	229,2705(1,5)	318(8,36)	136,5(0)	181,5(8,36)	5,605(0,28)	4,805(0,40)	4,115(0,49)	0,69(0,08)	138,5(7,83)	72,5(19,32)
175(3,13)	190(4,18)	226,27(0,52)	342(2,09)	138,6(2,19)	203,4(4,28)	1,15(0,05)	0,8205(0,05)	0,334(0,09)	0,4865(0,04)	94,5(51,70)	19(13,58)
403(7,31)	409(7,31)	215,784(2,09)	424(2,09)	243,6(6,58)	180,4(4,49)	4(0,21)	2,86(0,40)	2,315(0,20)	0,545(0,19)	40,5(38,12)	35,5(25,59)
390(2,09)	393(3,13)	212,787(0)	372(2,09)	137,55(1,10)	234,45(0,99)	5,79(0,46)	3,7325(0,54)	2,9355(0,09)	0,797(0,45)	65,5(43,35)	46,5(42,30)
103(1,04)	103(1,04)	19,4805(2,61)	131(11,49)	82,95(3,29)	48,05(8,20)	9,909(0,36)	0,834(0,05)	0,4325(0,01)	0,4015(0,07)	83,5(20,37)	47(6,27)
336(4,18)	359(3,13)	212,787(4,17)	281(19,84)	45,15(3,29)	235,85(16,55)	4,7395(0,52)	3,6235(0,52)	3,367(0,48)	0,2565(0,05)	29(6,27)	95,5(2,61)
333(28,20)	361(32,28)	239,2605(4,7)	465(7,31)	222,6(6,58)	242,4(0,73)	1,35(0,01)	0,956(0,03)	0,693(0,01)	0,263(0,04)	73(2,09)	23,5(20,37)
303(1,04)	303(1,04)	124,875(1,04)	289(1,04)	116,55(1,10)	172,45(2,14)	5,249(0,10)	3,446(0,02)	3,291(0,05)	0,155(0,02)	10,5(3,66)	56,5(14,10)
109(7,31)	109(7,31)	24,4755(1,57)	59(1,04)	29,4(2,19)	29,6(1,15)	8,187(0,13)	4,248(0,02)	0,897(0,03)	3,351(0,05)	78,5(13,06)	63(4,18)
137(1,04)	152(0)	21,978(2,09)	73(1,04)	31,5(2,19)	41,5(1,15)	3,25(0)	0,636(0,01)	0,345(0)	0,291(0,01)	55(17,76)	40,5(30,18)
445(7,31)	447(9,40)	187,3125(1,5)	433(7,31)	199,5(0)	233,5(7,31)	11,8065(0,57)	5,387(0,04)	5,2305(0,06)	0,1565(0,02)	46,5(30,81)	74(4,18)
364,66(11)	364,67(11)	167,166(0,50)	369,34(1)	233,1(3,15)	136,24(2,15)	6(0,15)	4,237(0,06)	3,887(0,08)	0,35(0,02)	107,34(50,50)	61,34(4)
425(17,76)	425(17,76)	185,814(2,09)	358(10,44)	208,95(3,29)	149,05(7,15)	12,85(0,99)	7,92(0,27)	6,085(0,74)	1,835(0,46)	136,5(10,97)	55,5(3,66)
385,5(3,09)	385,5(3,09)	201,79(0)	386(0)	222,25(2,16)	163,75(2,16)	4,6083(0,26)	4,005(0,03)	3,76(0,06)	0,245(0,03)	46,5(37,08)	82,5(4,70)
412(8,36)	412(8,36)	198,3015(2,6)	415(5,22)	224,7(0)	190,3(5,22)	5,65(0,57)	3,9(0,69)	3,635(0,51)	0,265(0,18)	146(4,18)	51(37,60)

411,54(6,23)	411,53(6,23)	222,47(4,15)	402,15(2,08)	215,17(1,09)	186,98(3,17)	8,52(0,06)	4,39(0,15)	3,287(0,04)	1,103(0,11)	103,77(10,38)	52,24(17,12)
108(0)	108(0)	31,4685(0,52)	153(3,13)	100,8(2,19)	52,2(0,94)	14,2405(1,04)	0,6255(0,07)	0,345(0,01)	0,2805(0,08)	103(13,58)	93(1,04)
380(2,09)	380(2,09)	206,793(5,22)	323(3,13)	84(0)	239(3,13)	9,724(2,74)	6,667(0,92)	6,0245(0,57)	0,6425(0,35)	95(6,27)	14,5(6,79)
381(1,04)	386(0)	266,2335(3,6)	528(0)	252(0)	276(0)	2,8825(0,75)	1,393(0,18)	0,578(0,05)	0,815(0,13)	42,5(27,68)	31(28,20)
427(1,04)	427(1,04)	172,3275(0,5)	348(2,09)	148,05(1,10)	199,95(3,19)	9,313(0,16)	6,16(0,06)	5,509(0,18)	0,651(0,24)	9(1,04)	59(19,84)
233(1,04)	233(1,04)	94,905(3,13)	157(1,04)	88,2(0)	68,8(1,04)	9,084(0,14)	4,267(0,42)	0,951(0,01)	3,316(0,42)	79(1,04)	81,5(8,88)
339(3,13)	350(4,18)	162,3375(0,5)	207(1,04)	111,3(0)	95,7(1,04)	4,624(0,06)	0,878(0,02)	0,461(0,01)	0,417(0,01)	64(59,53)	4(0)
490(4,18)	495(5,22)	202,797(1,04)	418(2,09)	207,9(2,19)	210,1(0,10)	11,865(0,36)	6,7405(0,19)	6,3935(0,47)	0,347(0,27)	87(25,07)	68,5(41,26)
271(1,04)	285(1,04)	137,862(1,04)	303(3,13)	172,2(2,19)	130,8(5,33)	4,05(0,16)	3,07(0,09)	2,35(0,14)	0,72(0,04)	139,5(5,74)	73,5(16,19)
405(9,40)	405(9,40)	185,814(0)	357(3,13)	218,4(4,39)	138,6(1,25)	12,915(0,14)	7,88(0,15)	7,01(0,15)	0,87(0)	76(69,98)	49(31,33)
298(4,18)	313(3,13)	210,785(1,04)	367(3,13)	176,4(2,19)	190,6(0,94)	10(0,21)	2,41(0,07)	1,785(0,04)	0,625(0,04)	134(6,27)	37(34,47)
409(3,13)	409(3,13)	207,792(7,30)	420(8,36)	249,9(10,97)	170,1(2,61)	4,2(0,10)	2,845(0,45)	2,41(0,16)	0,435(0,30)	142,5(6,79)	51,5(47,52)
390(2,09)	390(2,09)	216,783(1,04)	376(2,09)	219,45(1,10)	156,55(0,99)	7,561(0,34)	3,158(0,06)	2,8385(0,05)	0,3195(0,01)	101,5(54,83)	31(28,20)
90(4,18)	90(4,18)	16,983(1,04)	115(1,04)	77,7(2,19)	37,3(1,15)	15,6075(0,25)	0,8405(0,02)	0,494(0,01)	0,3465(0)	94(8,36)	50(1,04)
387(11,49)	387(11,49)	209,79(2,09)	320(4,18)	91,35(1,10)	228,65(5,27)	9,033(3,05)	6,184(1,11)	5,607(1)	0,577(0,10)	52,5(7,83)	108(1,04)
393(1,04)	428(0)	236,763(3,13)	472(8,36)	239,4(6,58)	232,6(1,78)	2,86(0,08)	2,1705(0,06)	1,61(0,06)	0,5605(0)	58(48,05)	2(0)
396(0)	396(0)	149,3505(0,5)	346(2,09)	143,85(1,10)	202,15(3,19)	11,966(0,58)	7,158(0,06)	6,017(0,22)	1,141(0,16)	31(1,04)	38(10,44)
173(1,04)	173(1,04)	63,936(0)	105(1,04)	66,15(1,10)	38,85(2,14)	9,9555(0,41)	4,276(0,07)	0,889(0,02)	3,387(0,05)	65(7,31)	102,5(0,52)
142(0)	169(15,67)	20,979(1,04)	88(4,18)	44,1(2,19)	43,9(1,98)	4,32(0,06)	0,845(0,04)	0,4445(0,02)	0,4005(0,02)	53,5(32,90)	41(7,31)
465(3,13)	465(13,13)	188,811(1,04)	402(2,09)	201,6(2,19)	200,4(4,28)	11,5555(1,77)	5,762(0,09)	4,938(0,30)	0,824(0,39)	47(14,62)	83(2,09)
354(6,27)	354(6,27)	168,3315(0,5)	353(5,22)	205,8(17,55)	147,2(22,77)	4,45(0,16)	3,16(0,06)	2,26(0,10)	0,9(0,04)	57(32,38)	27(2,09)
371(3,13)	381(3,13)	184,815(0)	361(1,04)	231(2,19)	130(1,15)	8,59(0,15)	7,635(0,08)	6,465(0,37)	1,17(0,29)	104,5(36,03)	68(7,31)
339(5,22)	353(5,22)	201,79(1,04)	394(6,27)	201,6(2,19)	192,4(4,07)	10,95(0,47)	4,745(0,22)	3,215(0,27)	1,53(0,04)	133(15,67)	45,5(34,99)
375(1,04)	396(4,18)	254,745(2,09)	349(7,31)	155,4(2,19)	193,6(9,50)	4,3(0,42)	2,83(0,47)	2,175(0,35)	0,655(0,12)	79,5(29,77)	52(8,36)
388(2,09)	398(2,09)	229,77(2,09)	373(5,22)	161,7(2,19)	211,3(3,03)	9,0625(0,41)	4,554(0,32)	3,331(0,10)	1,223(0,22)	26(13,58)	34,5(24,54)
83(1,04)	83(1,04)	24,975(2,09)	135(3,13)	72,45(5,48)	62,55(2,35)	10,8905(1,29)	0,5685(0,16)	0,4125(0,12)	0,156(0,05)	123,5(0,52)	25(5,22)
397(7,31)	402(8,36)	211,788(3,13)	347(3,13)	106,05(3,29)	240,95(0,16)	9,9405(0,75)	6,174(0,08)	5,2725(0,20)	0,9015(0,12)	72,5(46,48)	59,5(24,54)
371(1,04)	377(3,13)	226,2735(2,6)	442(6,27)	223,65(12,06)	218,35(5,80)	3,8425(0,77)	3,193(0,66)	2,6285(0,76)	0,5645(0,10)	58(30,29)	45,5(43,35)
372(4,18)	372(4,18)	159,84(3,13)	350(6,27)	145,95(3,29)	204,05(2,98)	7,298(0,04)	4,824(0,06)	4,5255(0,08)	0,2985(0,02)	26,5(22,46)	62,5(5,74)
154(2,09)	154(2,09)	62,937(1,04)	116(0)	71,4(4,39)	44,6(4,39)	8,427(0,36)	4,382(0,31)	0,745(0,02)	3,637(0,29)	67,5(16,19)	62(20,89)

149(1,04)	149(1,04)	35,4645(1,57)	100(4,18)	49,35(1,10)	50,65(5,27)	5,645(0,02)	0,813(0,02)	0,442(0,02)	0,371(0)	102(18,80)	59,5(48,57)
418(4,18)	429(5,22)	196,3035(2,6)	476(2,09)	211,05(5,48)	264,95(3,39)	11,892(0,34)	7,011(0,21)	6,6545(0,23)	0,3565(0,01)	46,5(13,06)	45,5(43,35)
311(15,67)	311(15,67)	158,3415(2,6)	356(4,18)	172,2(4,39)	183,8(0,21)	3,85(0,16)	2,93(0,16)	2,185(0,15)	0,745(0,01)	66(1,04)	68(18,80)
349(1,04)	356(2,09)	178,821(1,04)	353(3,13)	213,15(1,10)	139,85(2,04)	10,03(0,19)	8,14(0,02)	7,505(0,37)	0,635(0,35)	133,5(3,66)	82(2,09)
366(2,09)	381,5(3,66)	193,81(1,04)	389(1,04)	214,2(10,97)	174,8(9,92)	5,1(0,84)	3,035(0,05)	2,665(0,08)	0,37(0,03)	78(62,67)	87,5(9,92)
441(15,67)	441(15,67)	187,812(2,09)	372(13,53)	207,9(4,39)	164,1(16,92)	4,05(0,26)	2,81(0,39)	2,18(0,28)	0,63(0,10)	118,5(14,10)	60(14,62)
348(4,18)	348(4,18)	202,797(1,04)	324(2,09)	172,2(0)	151,8(2,09)	5,7795(0,82)	3,5885(0,65)	3,114(0,81)	0,4745(0,16)	64(35,51)	72,5(15,14)
115(3,13)	115(3,13)	36,4635(2,61)	174(12,53)	113,4(4,39)	60,6(16,92)	8,945(0,30)	0,531(0,01)	0,311(0,01)	0,22(0)	88,5(0,52)	40(7,31)
410(8,36)	410(8,36)	200,799(4,17)	350(25,07)	72,45(9,87)	277,55(15,20)	6,8005(0,48)	5,8255(0,94)	5,6595(1,02)	0,166(0,08)	83(27,16)	56,5(30,81)
349(9,40)	377(9,40)	175,824(0)	364(2,09)	176,4(4,39)	187,6(2,30)	1,095(0,02)	0,5955(0,05)	0,15(0,01)	0,4455(0,04)	87(33,42)	49(49,09)
344(2,09)	344(2,09)	165,3345(1,5)	307(1,04)	179,55(3,29)	127,45(2,25)	12,655(0,13)	6,888(0,28)	6,535(0,32)	0,353(0,05)	41,5(0,52)	50,5(29,77)
143(1,04)	143(1,04)	53,4465(0,52)	95(1,04)	52,5(2,19)	42,5(1,15)	7,932(0,28)	4,985(0,06)	0,906(0,06)	4,079(0,13)	57,5(4,70)	90,5(14,10)
217(1,04)	217(1,04)	91,4085(0,52)	133(3,13)	71,4(2,19)	61,6(0,94)	5,718(0,11)	0,895(0,07)	0,472(0)	0,423(0,08)	25(19,84)	83,5(14,10)
419(3,13)	426(4,18)	169,83(2,09)	339(1,04)	186,9(2,19)	152,1(1,15)	14,761(1,12)	7,5995(0,07)	6,958(0,17)	0,6415(0,11)	46,5(22,46)	56,5(8,88)
344(10,44)	344(10,44)	156,843(12,5)	365(19,84)	174,3(36,32)	190,7(6,48)	5,5(0,21)	3,96(0,13)	3,65(0,11)	0,31(0,01)	65,5(30,81)	51(26,11)
298(2,09)	303(3,13)	162,837(1,04)	298(4,18)	197,4(8,77)	100,6(12,95)	8,095(0,24)	6,835(0,05)	6,38(0,14)	0,455(0,18)	128(25,07)	18,5(13,06)
366(4,18)	378(2,09)	190,31(1,57)	380(2,09)	214,2(6,58)	165,8(4,49)	4,9(0,10)	3,82(0,07)	3,245(0,12)	0,575(0,05)	134(1,04)	64,5(2,61)
452(8,36)	452(8,36)	197,802(0)	380(14,62)	215,25(14,26)	164,75(28,88)	4,8(0,63)	3,745(0,38)	3,06(0,80)	0,685(0,42)	107,5(20,37)	42(3,13)
372(2,09)	372(2,09)	212,787(2,09)	367(3,13)	180,6(2,19)	186,4(0,94)	4,6285(0,04)	3,565(0,31)	3,0315(0,07)	0,5335(0,24)	92,5(20,37)	82,5(28,72)
122(6,27)	122(6,27)	35,4645(0,52)	154(10,44)	114,45(7,68)	39,55(2,77)	12,5685(1,04)	0,723(0,06)	0,343(0,02)	0,38(0,08)	108(2,09)	67,5(1,57)
442(31,33)	442(31,33)	202,2975(8,8)	310(6,27)	80,85(9,87)	229,15(3,60)	8,8165(0,47)	5,769(0,22)	5,146(0,04)	0,623(0,18)	72(26,11)	79(20,89)
340(2,09)	357(3,13)	184,815(5,22)	377(3,13)	187,95(12,06)	189,05(15,20)	1,815(0,08)	1,5795(0,05)	1,1495(0,04)	0,43(0,01)	123(6,27)	16(7,31)
367(1,04)	367(1,04)	171,3285(3,6)	336(6,27)	199,5(6,58)	136,5(0,31)	10,05(0,23)	6,584(0,24)	6,227(0,12)	0,357(0,11)	2,5(0,52)	79(16,71)
146(4,18)	146(4,18)	52,4475(3,65)	87(1,04)	54,6(0)	32,4(1,04)	10,002(0,37)	5,188(0,04)	0,822(0,01)	4,366(0,03)	114,5(0,52)	16,5(0,52)
245(1,04)	250(0)	106,893(2,09)	154(2,09)	76,65(3,29)	77,35(5,38)	5,165(0,04)	1,0055(0,02)	0,656(0,01)	0,3495(0,01)	75(21,93)	57(44,99)
422(2,09)	428,5(1,57)	174,825(1,04)	402(2,09)	196,35(1,10)	205,65(3,19)	12,593(0,13)	6,885(0,04)	6,0955(0,45)	0,7895(0,41)	86,5(7,83)	52(51,18)
336(4,18)	336(4,18)	167,3325(3,6)	362(0)	180,6(8,77)	181,4(8,77)	4,9(0,21)	3,775(0,19)	3,405(0,14)	0,37(0,05)	23(8,36)	83,5(4,70)
352(0)	357(1,04)	188,3115(0,5)	353(1,04)	218,4(2,19)	134,6(1,15)	7,685(0,07)	6,61(0,07)	6,08(0,16)	0,53(0,08)	94(6,27)	65(10,44)
337(7,31)	352(8,36)	206,29(0,52)	401(1,04)	224,7(2,19)	176,3(1,15)	7,6(0,21)	3,06(0,02)	2,71(0)	0,35(0,02)	152(1,04)	43(15,67)
358(2,09)	365(1,04)	229,77(4,17)	371(1,04)	204,75(9,87)	166,25(10,91)	4,75(0,47)	3,72(0,42)	3,16(0,87)	0,56(0,45)	108,5(17,23)	39(34,47)

367(1,04)	367(1,04)	204,795(1,04)	287(5,22)	127,05(3,29)	159,95(1,93)	5,1895(0,09)	3,894(0,10)	3,332(0,12)	0,562(0,02)	77(19,84)	54(16,71)
116(12,53)	116(12,53)	35,964(2,09)	152(6,27)	109,2(8,77)	42,8(2,51)	8,666(0,31)	0,919(0,03)	0,4875(0,02)	0,4315(0,06)	65(9,40)	45(10,44)
404(2,09)	411(3,13)	227,2725(2,6)	375(17,76)	96,6(8,77)	278,4(8,98)	7,004(0,13)	2,8735(0,10)	2,5895(0,08)	0,284(0,17)	24(4,18)	50,5(48,57)
321(3,13)	326(2,09)	187,812(10,4)	368(0)	181,65(7,68)	186,35(7,68)	3,0175(0,03)	1,12(0,12)	0,822(0,03)	0,298(0,09)	84(39,69)	29(5,22)
341(1,04)	341(1,04)	172,3275(1,5)	332(2,09)	202,65(3,29)	129,35(1,20)	7,374(0,09)	4,642(0,13)	4,277(0,06)	0,365(0,07)	23(20,89)	61,5(45,43)
144(0)	144(0)	55,4445(1,57)	93(1,04)	49,35(1,10)	43,65(0,05)	7,336(0,24)	4,323(0,25)	0,815(0,05)	3,508(0,20)	90(9,40)	7,5(2,61)
289(1,04)	295(1,04)	130,869(2,09)	174(2,09)	74,55(1,10)	99,45(0,99)	4,269(0,06)	0,545(0,02)	0,329(0,05)	0,216(0,03)	35,5(15,14)	30,5(13,06)
413(1,04)	425(5,22)	186,813(1,04)	404(0)	192,15(1,10)	211,85(1,10)	11,8235(0,55)	6,0985(0,37)	5,801(0,32)	0,2975(0,05)	28(9,40)	74,5(3,66)
**<0.0001**	**<0.0001**	**<0.0001**	**<0.0001**	**<0.0001**	**<0.0001**	**<0.0001**	**<0.0001**	**<0.0001**	**<0.0001**	**<0.0001**	**<0.0001**

Abbreviations: Abtemt: ambient temperature, Wtemp: water temperature, Cond: conductivity, TDS: total dissolved solids, DO: dissolved oxygen, Turb: turbidity, TSS: total suspended solids, BOD: biochemical oxygen demand, COD: chemical oxygen demand, Free CO_2: free carbon dioxide, CO_3Alk: phenolphthalein alkalinity, HCO3 Alk: methyl orange alkalinity, Talk: total alkalinity, Cl: chloride, TH: total hardness, CaH: calcium hardness, MgH: magnesium hardness, NO_3-N: nitrate nitrogen, TP: total phosphorous, Ortho P: ortho phosphorus, OrgP: organic phosphorus, TCC: total coliform, TEC: total *E. Coli*. NA: missing.

Table S10. Correlation analysis between antibiotic and water quality parameters of the Kshipra river in India over a 3-year period.

**Parameters**	**Sulfamethoxazole. (µg/L)**		**Ofloxacin. (µg/L)**		**Norfloxacin (µg/L)**		**Metronidazole. (µg/L)**		**Ampicillin**		**Cefotaxime**		**Co-trimoxazole**	
	**RHO**	**P-value**	**RHO**	**P-value**	**RHO**	**P-value**	**RHO**	**P-value**	**RHO**	**P-value**	**RHO**	**P-value**	**RHO**	**P-value**
Abtemt.(°C)	0,139	0.00061	0,061	0,063	0,024	0,404	-0,254	<0.0001	0,227	<0.0001	0,172	<0.0001	0,113	0.00137
Wtemp.(°C)	0,138	0.00505	0,173	<0.0001	0,149	<0.0001	-0,133	<0.0001	0,189	<0.0001	0,169	<0.0001	0,108	0.00163
PH	-0,034	0.06013	0,053	0,154	0,071	0,055	-0,628	<0.0001	0,183	<0.0001	0,082	0.00793	0,061	0.07519
Cond. (µS/cm)	0,299	<0.0001	0,001	0,406	0,137	0,005	-0,621	<0.0001	-0,033	0.27444	-0,078	0.01465	-0,008	0.77245
TDS (mg/L)	-0,167	<0.0001	0,007	0,608	0,124	0,009	-0,633	<0.0001	0,174	<0.0001	0,111	0.00012	0,145	<0.0001
DO (mg/L)	-0,243	0.00553	-0,066	0,189	-0,139	0,001	-0,024	0,845	0,141	0.08257	0,102	0.01955	0,109	0.41384
Turb. (NTU)	-0,076	0.00021	-0,167	<0.0001	-0,164	<0.0001	0,895	<0.0001	-0,056	0.05995	-0,102	0.00319	-0,010	0.42867

TSS (mg/L)	0,173	<0.0001	-0,130	0,001	-0,131	0,001	0,874	<0.0001	-0,024	0.38449	0,034	0.524	-0,072	0.03804
BOD (mg/L)	0,073	0.00416	0,038	0,694	0,093	0,064	-0,303	<0.0001	0,006	0.91663	0,007	0.86381	-0,062	0.08704
COD (mg/L)	-0,085	0.00916	0,059	0,402	0,118	0,018	-0,255	<0.0001	0,025	0.47872	-0,011	0.80316	-0,035	0.3591
Free CO_2 (mg/L)	0,051	0.54589	0,047	0,096	0,164	<0.0001	0,246	<0.0001	-0,031	0.58909	0,025	0.32149	0,045	0.37952
CO_3Alk (mg/L)	0,002	0.95967	0,031	0,724	-0,154	<0.0001	-0,265	<0.0001	0,157	<0.0001	0,140	<0.0001	0,108	0.0015
HCO3 Alk (mg/L)	-0,243	<0.0001	-0,037	0,194	0,041	0,425	-0,654	<0.0001	0,036	0.11137	0,069	0.01339	-0,020	0.5897
Talk. (mg/L)	0,482	<0.0001	-0,050	0,106	0,024	0,720	-0,671	<0.0001	-0,007	0.72812	-0,018	0.45208	0,012	0.71869
Cl (mg/L)	0,243	<0.0001	-0,110	0,001	-0,035	0,208	-0,642	<0.0001	0,106	0.00663	0,066	0.06794	0,098	0.00218
TH (mg/L)	-0,144	<0.0001	-0,065	0,042	0,104	0,011	-0,507	<0.0001	0,157	<0.0001	0,160	<0.0001	0,106	0.00112
CaH (mg/L)	0,158	<0.0001	0,010	0,590	0,186	<0.0001	-0,281	<0.0001	0,080	0.01099	0,075	0.02272	0,035	0.28358
MgH (mg/L)	0,245	<0.0001	-0,115	0,006	-0,004	0,848	-0,561	<0.0001	0,006	0.95695	0,006	0.72679	-0,028	0.74347
NO_3-N (mg/L)	-0,419	<0.0001	-0,142	<0.0001	-0,142	<0.0001	0,522	<0.0001	0,003	0.66422	0,064	0.01356	-0,011	0.87056
TP (mg/L)	0,197	<0.0001	-0,007	0,030	0,127	0,497	-0,401	<0.0001	-0,020	0.63541	-0,049	0.18104	0,000	0.96087
OrthoP (mg/L)	-0,178	<0.0001	-0,007	0,034	0,129	0,426	-0,390	<0.0001	-0,136	<0.0001	-0,104	0.00119	-0,022	0.41937
OrgP (mg/L)	0,148	<0.0001	-0,004	0,250	0,028	0,699	-0,190	<0.0001	0,025	0.72434	-0,086	0.00584	0,054	0.11093
CFU/100ml (TCC)	-0,285	<0.0001	-0,067	0,014	-0,110	<0.0001	0,066	0,362	0,001	0.82877	0,004	0.83701	-0,017	0.48874
CFU/100ml (TEC)	0,401	<0.0001	0,064	0,158	-0,006	0,688	0,029	0,616	0,024	0.58067	-0,012	0.5961	0,007	0.85783

Cont. Table S10. Correlation analysis between antibiotic and water quality parameters of the Kshipra river in India over a 3-year period.

Parameters	Ceftazidime		Cefepime		NalidixicAcid		Ciprofloxacin		Nitrofurantoin		Gentamicin		Meropenem	
	RHO	P-value	RHO	P-value	RHO	P-value	RHO	P-value	RHO	P-value	RHO	P-value	RHO	P-value
Abtemt.(°C)	0,212	<0.0001	0,407	<0.0001	0,120	0.00033	0,157	<0.0001	0,203	<0.0001	0,324	<0.0001	0,299	<0.0001
Wtemp.(°C)	0,239	<0.0001	0,394	<0.0001	0,125	<0.0001	0,157	<0.0001	0,177	<0.0001	0,297	<0.0001	0,341	<0.0001
PH	0,060	0.02946	0,034	0.15139	0,039	0.12104	0,053	0.03734	0,093	0.00371	0,036	0.07568	-0,001	0.45737

Cond. (µS/cm)	-0,052	0.06874	-0,180	<0.0001	-0,067	0.02699	-0,054	0.05383	0,008	0.95704	-0,062	0.02364	-0,006	0.46296
TDS (mg/L)	0,106	<0.0001	0,203	<0.0001	0,116	<0.0001	0,134	<0.0001	0,135	<0.0001	0,192	<0.0001	-0,011	0.11615
DO (mg/L)	0,074	0.00043	0,209	0.00597	0,101	0.01956	0,112	0.00156	0,106	0.11938	0,177	<0.0001	-0,038	<0.0001
Turb. (NTU)	-0,143	0.00013	-0,205	0.00027	-0,032	<0.0001	-0,036	<0.0001	0,028	0.0026	-0,048	<0.0001	-0,121	<0.0001
TSS (mg/L)	0,081	0.06599	0,085	0.04561	0,000	0.71637	-0,002	0.59095	-0,027	0.30227	0,017	0.87308	0,159	<0.0001
BOD (mg/L)	0,079	0.08626	0,121	0.00356	-0,033	0.15704	0,010	0.73436	0,020	0.86134	0,051	0.63828	0,204	<0.0001
COD (mg/L)	0,032	0.33053	0,060	0.0828	-0,034	0.36334	0,017	0.58422	0,010	0.73889	0,028	0.42299	0,110	0.00157
Free CO2 (mg/L)	0,055	0.03861	0,054	0.04444	-0,046	0.52333	0,007	0.3627	0,008	0.56678	0,033	0.02627	-0,013	0.42922
CO3 Alk (mg/L)	0,157	<0.0001	0,272	<0.0001	0,066	0.03724	0,140	<0.0001	0,249	<0.0001	0,378	<0.0001	0,186	<0.0001
HCO3 Alk (mg/L)	0,097	0.00037	0,050	0.01373	0,013	0.11105	0,041	0.00771	-0,028	0.70546	-0,002	0.01051	-0,018	0.06077
Talk. (mg/L)	0,005	0.8668	0,023	0.7289	-0,046	0.11453	-0,054	0.06078	0,002	0.88025	0,014	0.9085	0,068	0.14781
Cl (mg/L)	0,079	0.03303	0,174	<0.0001	0,041	0.39918	0,092	0.03214	0,227	<0.0001	0,304	<0.0001	0,173	<0.0001
TH (mg/L)	0,198	<0.0001	0,169	<0.0001	0,089	0.00151	0,132	<0.0001	0,108	0.00016	0,186	<0.0001	0,087	0.00015
CaH (mg/L)	0,125	0.00017	0,063	0.03031	0,033	0.16812	0,015	0.26693	-0,017	0.89646	0,003	0.2596	0,028	0.08553
MgH (mg/L)	0,061	0.07048	0,031	0.37438	0,023	0.63354	-0,002	0.81587	0,003	0.94956	0,017	0.78569	0,129	0.0012
NO3-N (mg/L)	0,069	0.01083	-0,008	0.57333	-0,006	0.45303	0,013	0.13695	-0,073	0.08353	-0,030	0.62674	-0,037	0.78007
TP (mg/L)	-0,067	0.06626	-0,070	0.05756	0,019	0.50698	-0,051	0.20527	-0,017	0.73081	-0,098	0.01657	-0,139	0.00021
OrthoP (mg/L)	-0,155	<0.0001	-0,213	<0.0001	-0,051	0.09297	-0,096	0.00248	-0,141	<0.0001	-0,275	<0.0001	-0,213	<0.0001
OrgP (mg/L)	-0,160	<0.0001	-0,100	0.00072	-0,027	0.16228	-0,028	0.10322	0,090	0.05721	0,039	0.768	-0,036	0.03047
CFU/100ml (TCC)	-0,054	0.18508	-0,105	0.0082	-0,029	0.62819	-0,064	0.17912	-0,104	0.00854	-0,168	<0.0001	-0,142	0.00068
CFU/100ml (TEC)	0,033	0.50513	-0,037	0.1865	-0,013	0.53144	-0,043	0.12309	-0,022	0.42386	-0,075	0.01122	0,069	0.13074

Cont. Table S10. Correlation analysis between antibiotic and water quality parameters of the Kshipra river in India over a 3-year period.

**Parameters**	**Amikasin**		**Tetracycline**		**Tigecycline**		**Imipenem**		**Sulfamethiazole**	

	RHO	P-value	RHO	P-value	RHO	P-value	RHO	P-value	RHO	P-value
Abtemt.(°C)	0,384	<0.0001	0,177	<0.0001	0,085	0.0087	0,001	0.73946	0,124	0,0012
Wtemp.(°C)	0,372	<0.0001	0,143	<0.0001	0,094	0.00035	-0,027	0.70848	-0,063	0,26336
PH	0,048	0.0324	0,018	0.36118	-0,092	0.06421	0,013	0.2575	0,031	0,2093
Cond. (µS/cm)	-0,017	0.28668	-0,051	0.0825	0,016	0.95362	0,092	0.04407	0,004	0,8624
TDS (mg/L)	0,137	<0.0001	0,201	<0.0001	-0,055	0.84761	-0,019	0.19096	0,100	0,00018
DO (mg/L)	0,100	<0.0001	0,182	0.10852	-0,043	<0.0001	-0,056	<0.0001	0,077	0,21339
Turb. (NTU)	-0,047	<0.0001	-0,033	0.00132	-0,055	<0.0001	0,048	<0.0001	-0,026	0,00116
TSS (mg/L)	0,061	0.28618	-0,087	0.00541	0,064	0.22612	0,022	0.99116	-0,050	0,08787
BOD (mg/L)	0,121	0.01365	-0,049	0.07628	0,075	0.18141	0,065	0.37068	-0,037	0,16316
COD (mg/L)	0,092	0.00854	0,051	0.13887	0,032	0.33777	0,000	0.96324	-0,025	0,53282
Free CO2 (mg/L)	-0,009	0.37132	0,058	0.05435	0,046	0.01723	0,023	0.03994	0,105	0,0039
CO3 Alk (mg/L)	0,360	<0.0001	0,141	<0.0001	0,091	0.00317	0,170	<0.0001	0,074	0,02265
HCO3 Alk (mg/L)	-0,027	0.10347	0,010	0.16853	-0,083	0.99132	0,032	0.00059	0,105	0,00085
Talk. (mg/L)	0,049	0.38531	0,001	0.84627	0,020	0.90564	-0,016	0.33512	-0,019	0,46605
Cl (mg/L)	0,362	<0.0001	0,103	0.00823	0,085	0.05745	0,147	0.00129	-0,011	0,80691
TH (mg/L)	0,213	<0.0001	0,094	0.00078	-0,129	0.02771	0,101	<0.0001	0,099	0,00035
CaH (mg/L)	0,008	0.21627	0,019	0.31655	-0,105	0.04444	0,001	0.28334	0,072	0,01322
MgH (mg/L)	0,041	0.5322	-0,050	0.16639	0,127	0.00129	0,094	0.04592	-0,002	0,91565
NO3-N (mg/L)	-0,044	0.93134	0,049	0.04859	-0,112	0.03361	0,019	0.07077	0,059	0,0244
TP (mg/L)	-0,152	<0.0001	-0,041	0.28809	-0,024	0.63214	-0,055	0.21367	0,051	0,12837
OrthoP (mg/L)	-0,310	<0.0001	-0,063	0.04132	-0,008	0.59449	-0,160	<0.0001	0,000	0,7846
OrgP (mg/L)	-0,008	0.12732	0,047	0.45631	0,156	0.00148	0,073	0.44849	-0,020	0,32318
CFU/100ml (TCC)	-0,231	<0.0001	-0,006	0.91341	-0,073	0.10721	-0,098	0.0301	0,034	0,29553
CFU/100ml (TEC)	-0,026	0.23221	-0,054	0.08335	0,019	0.91871	0,065	0.16391	0,005	0,94325

Abbreviations: Abtemt: ambient temperature, Wtemp: water temperature, Cond: conductivity, TDS: total dissolved solids, DO: dissolved oxygen, Turb: turbidity, TSS: total suspended solids, BOD: biochemical oxygen demand, COD: chemical oxygen demand, Free CO_2: free carbon dioxide, CO_3Alk: phenolphthalein alkalinity, HCO3 Alk: methyl orange alkalinity, Talk: total alkalinity, Cl: chloride, TH: total hardness, CaH: calcium hardness, MgH: magnesium hardness, NO_3-N: nitrate nitrogen, TP: total phosphorous, Ortho P: ortho phosphorus, OrgP: organic phosphorus, TCC: total coliform, TEC: total *E. Coli.* NA: missing.

Table S11. Correlation analysis between antibiotics and sediment quality parameters of the Kshipra river in India over a 3-year period.

Parameters	Ampicillin		Cefotaxime		Ceftazidime		Cefepime		NalidixicAcid	
	RHO	P-value	RHO	P-value	RHO	P-value	RHO	P-value	RHO	P-value
pH	0,05	0,42869	0,03	0,79822	0,04	0,50552	0,04	0,44291	0,00	0,84333
SolCO3 (mg/kg)	-0,17	0,00154	-0,09	0,13269	-0,16	0,00168	-0,17	0,00058	-0,04	0,48679
Cl (mg/kg)	-0,01	0,94491	-0,07	0,19652	-0,08	0,11	-0,18	0,00037	0,06	0,23359
NO3-N (mg/g)	-0,02	0,79086	0,00	0,94492	-0,07	0,19569	-0,07	0,18242	0,00	8,85E-01
AP (mg/g)	0,01	0,7902	0,04	0,38961	-0,04	0,47275	-0,05	0,31029	0,02	0,64937
OM (%)	-0,07	0,16112	-0,01	0,91482	-0,01	0,95781	0,08	0,09691	-0,02	0,81298
CFU/100 (TCC)	-0,06	0,2173	-0,06	0,22038	0,02	0,65164	0,03	0,52075	0,01	0,89677
CFU/100ml (TEC)	0,00	0,92734	-0,07	0,22374	-0,12	0,01987	0,04	4,87E-01	0,06	0,23009
Parameters	Ciprofloxacin		Nitrofurantoin		Gentamicin		Amikacin		Tetracycline	
	RHO	P-value	RHO	P-value	RHO	P-value	RHO	P-value	RHO	P-value
pH	-0,03	0,39552	0,04	0,00894	-0,03	0,68812	0,04	0,02481	0,03	0,58313
SolCO3 (mg/kg)	0,02	0,51406	0,03	1,70E-04	-0,11	0,026	-0,15	0,02165	-0,07	0,17288
Cl (mg/kg)	-0,04	0,55503	-0,04	8,89E-03	-0,07	0,14551	-0,11	0,1115	-0,03	0,5746
NO3-N (mg/g)	-0,01	0,97875	-0,21	0,0935	-0,37	<0.0001	-0,32	<0.0001	0,03	0,53656
AP (mg/g)	0,02	5,75E-01	-0,19	0,01848	-0,36	<0.0001	-0,32	0,0978	0,07	0,18098
OM (%)	0,02	0,61772	0,00	0,17527	0,02	0,79509	-0,01	0,67103	0,01	0,87704
CFU/100 (TCC)	-0,01	0,88717	0,02	0,34598	0,09	0,06992	0,05	0,17704	-0,10	0,04904
CFU/100ml (TEC)	-0,03	5,63E-01	0,03	0,05089	-0,05	0,25808	-0,05	0,20221	0,03	0,50017
Parameters	Tigecycline		Imipenem		Meropenem		Co-trimoxazole		Sulfamethizole	
	RHO	P-value	RHO	P-value	RHO	P-value	RHO	P-value	RHO	P-value
pH	0,05	0,30795	-0,22	<0.0001	0,01	0,68273	0,04	0,50874	0,06	0,36545
SolCO3 (mg/kg)	-0,03	0,50471	-0,03	0,47199	-0,25	<0.0001	-0,03	0,61106	0,02	0,53907
Cl (mg/kg)	-0,04	0,41819	-0,03	0,52388	-0,28	<0.0001	-0,03	0,53194	-0,02	0,82933
NO3-N (mg/g)	-0,02	0,63341	-0,37	<0.0001	-0,08	0,10867	0,07	0,17333	0,02	0,6008
AP (mg/g)	0,01	0,86787	-0,34	<0.0001	-0,05	0,29909	0,10	0,04687	0,04	0,42687

OM (%)	0,21	<0.0001	-0,13	0,0102	0,07	0,16321	-0,03	0,58058	0,02	0,70971
CFU/100 (TCC)	-0,10	0,03955	0,00	0,94087	-0,08	0,12499	-0,06	0,21664	-0,06	0,23411
CFU/100ml (TEC)	-0,01	0,79305	-0,17	0,00074	0,03	0,58735	0,02	0,622	0,02	0,61925

Abbreviations: SolCO3: soluble bicarbonate, Cl: chloride, NO_3-N: nitrate nitrogen, AP: available phosphorus, OM: organic matter, TCC: total coliform, TEC: total *E. Coli.*

Table S12. Water quality standards.

Water quality parameter	Standard	Source
pH	5 – 9	[1]
Water temperature (°C)	32	[2]
Conductivity (µS/cm)	1000	[1]
Total dissolved solids (mg/L)	500	[2]
Turbidity (NTU)	10	[2]
Total Alkalinity (mg/L)	30 to 90	[1]
Chloride(mg/L)	230	[1]
Total Hardness (mg/L)	300	[2]
Nitrate Nitrogen (mg/L)	10	[1]
Total Phosphorus (mg/L)	0.01	[1]
Sulphate (mg/L)	400	[3]
Dissolved oxygen (mg/L)	5	[3]
Biochemical Oxygen Demand (mg/L)	8	[2]
Chemical Oxygen Demand (mg/L)	20	[2]
Total Coliform CFU/100 ml	50 or less	[4]
Total *E. coli* CFU/100 ml	Shall not be detectable	[4]

References

[1] EPA, 'National Recommended Water Quality Criteria', 2009, [Online]. Available: file:///C:/Users/nadhan/Downloads/644569.pdf.

[2] EPA, 'Brungs, W.S. and B.R. Jones. 1977. Temperature Criteria for Freshwater Fish: Protocols and Procedures. EPA-600/3-77-061. Environ. Research Lab, Ecological Resources Service, U.S. Environmental Protection Agency, Office of Research and Development, Duluth, MN.', 1977, [Online]. Available: https://nepis.epa.gov/Exe/ZyNET.exe/9100T3ND.TXT?ZyActionD=ZyDocument&Client=EPA&Index=1976+Thru+1980&Docs=&Query=&Time=&EndTime=&SearchMethod=1&TocRestrict=n&Toc=&TocEntry=&QField=&QFieldYear=&QFieldMonth=&QFieldDay=&IntQFieldOp=0&ExtQFieldOp=0&XmlQuery=&File=D%3A%5Czyfiles%5CIndex%20Data%5C76thru80%5CTxt%5C00000018%5C9100T3ND.txt&User=ANONYMOUS&Password=anonymous&SortMethod=h%7C-&MaximumDocuments=1&FuzzyDegree=0&ImageQuality=r75g8/r75g8/x150y150g16/i425&Display=hpfr&DefSeekPage=x&SearchBack=ZyActionL&Back=ZyActionS&BackDesc=Results%20page&MaximumPages=1&ZyEntry=1&SeekPage=x&ZyPURL.

[3] WHO, ‘Standard for Drinking Water Quality.World Health Organization, Geneva.’, 1997.

[4] WHO, ‘Drinking water Specification-Indian Standard IS10500:2012(second revision)’, 2005, [Online]. Available: http://www.indiawaterportal.org/sites/indiawaterportal.org/files/drinking_water_standards_bis_10500_2004_by_bis.pdf.
